# Cerebral Injury and Impaired Brain Metabolism in Patients With Left Ventricular Assist Device and Chronic Heart Failure —An Interdisciplinary Cognition, Blood‐Based Biomarker and Brain Metabolism Assessment

**DOI:** 10.1161/JAHA.125.043727

**Published:** 2026-09-09

**Authors:** Martin S. Jungclaus, Lorenzo Barba, Markus Otto, Florian Wilke, Arjang Ruhparwar, Jasmin S. Hanke, Jan D. Schmitto, Dominik Berliner, Thorsten Derlin, Rudolf A. Werner, Frank M. Bengel, Karin Weissenborn, Kai G. Kahl, Julia Hoffmeister, Georg Berding, Hans Worthmann, Desiree Weiberg, Maria M. Gabriel

**Affiliations:** ^1^ Department of Neurology Hannover Medical School Hannover Germany; ^2^ Department of Neurology Martin‐Luther‐University Halle‐Wittenberg Halle (Saale) Germany; ^3^ Department of Nuclear Medicine Hannover Medical School Hannover Germany; ^4^ Department of Cardiac, Thoracic, Transplantation and Vascular Surgery Hannover Medical School Hannover Germany; ^5^ Department of Cardiology and Angiology Hannover Medical School Hannover Germany; ^6^ Institute of Radiology, Nuclear Medicine, and Molecular Imaging, Heart and Diabetes Center North Rhine‐Westphalia, Ruhr University Bochum and Medical Faculty OWL (University Bielefeld) Bad Oeynhausen Germany; ^7^ Department of Nuclear Medicine Ludwig‐Maximilians‐University of Munich Munich Germany; ^8^ Department of Psychiatry, Social Psychiatry and Psychotherapy Hannover Medical School Hannover Germany

**Keywords:** brain metabolism, cognitive impairment, FDG‐PET, fluid biomarkers, left ventricular assist device, Heart Failure, Imaging, Biomarkers, Cerebrovascular Disease/Stroke, Cognitive Impairment

## Abstract

**Background:**

Chronic heart failure is linked to cognitive dysfunction. Left ventricular assist devices (LVAD) potentially ameliorate cognition, but continuous flow profiles might result in long‐term progression. In this cross‐sectional pilot study, we aimed to investigate the association between LVAD therapy and neuronal impairment measured by cognitive performance, blood‐based biomarkers of neuronal injury and ^18^F‐fluorodeoxyglucose‐positron emission tomography/computed tomography‐derived brain metabolism.

**Methods:**

We prospectively enrolled 19 patients with LVAD and 13 patients with advanced chronic heart failure receiving best medical treatment. Cognitive performance was assessed using the Montreal Cognitive Assessment and Frontal Assessment Battery. Serum levels of NfL (neurofilament light chain), pTau181 (phosphorylated Tau protein at threonine 181), and GFAP (glial fibrillary acidic protein) were measured by immunoassay. Brain metabolism was assessed using ^18^F‐fluorodeoxyglucose‐positron emission tomography/computed tomography by statistical parametric mapping analyses compared with 16 healthy controls (*P*<0.001 uncorrected).

**Results:**

Of all patients, 69% showed cognitive dysfunction, 5 patients had evidence for frontotemporal impairment. Median neurochemical biomarker concentrations were elevated in the whole cohort (NfL 45.7 pg/mL [26.9–80.2]; pTau181 23.1 pg/mL [18.1–40.1]; GFAP 191.3 pg/mL [126.3–251.7]). Both groups did not differ regarding cognitive performance and biomarker levels. The uncorrected statistical parametric mapping analyses indicated frontotemporally accentuated hypometabolism in both groups compared with healthy controls. Patients with LVAD revealed more hypometabolic volumes in single‐subject analyses (LVAD 1.03 mL (0–7.34); chronic heart failure 0 mL (0–0.01), *P*<0.003).

**Conclusions:**

In this exploratory study, patients with chronic heart failure and patients with LVAD exhibited impaired cognitive performance and elevated biomarker levels of neuronal injury. ^18^F‐fluorodeoxyglucose‐positron emission tomography/computed tomography results of uncorrected thresholds suggested subtle frontal hypometabolism. Neurochemical biomarkers and ^18^F‐fluorodeoxyglucose‐positron emission tomographyappear promising for further investigation of cerebral health in patients with heart failure.

## Graphical abstract

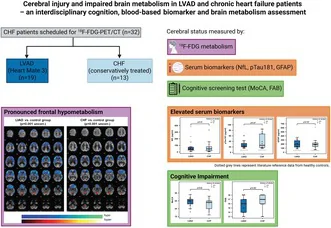

**Nonstandard Abbreviations and Acronyms**
^18^F‐FDG‐PET/CT: ^18^F‐fluorodeoxyglucose‐positron emission tomography/ computed tomography
AV: aortic valve
CHF: chronic heart failure
FWE: family‐wise error
GFAP: glial fibrillary acidic protein
HM3: HeartMate 3
HVAD: HeartWare HVAD System
NfL: neurofilament light chain protein
pTau181: phosphorylated Tau protein at threonine 181
SSVCG: single subject versus control group
THV: total hypometabolic volume

**Clinical Perspective**

**What Is New?**

- For the first time neuronal health was assessed in patients with chronic heart failure with and without left ventricular assist device therapy using ^18^F‐fluorodeoxyglucose‐positron emission tomography/computed tomography, neurocognitive testing and neurodegenerative serum biomarkers (ie, NfL [neurofilament light chain], pTau181 [phosphorylated Tau protein at threonine 181], and GFAP [glial fibrillary acidic protein]).
- Our uncorrected imaging results suggest a frontotemporally pronounced cerebral hypometabolism in left ventricular assist device and conservatively treated patients with chronic heart failure, with similarly diminished cognitive capabilities and elevated neurodegenerative marker levels.

**What Are the Clinical Implications?**

- The results highlight the necessity to develop tools for prevention of cognitive decline in patients with chronic heart failure in long‐term conservative as well as mechanically supported heart failure care; particular attention should be directed towards the most frequently affected domains short‐ and long‐term memory, verbal fluency, and attention.

The ongoing shortage of available donor hearts has led to the increased use of left ventricular assist devices (LVAD) as long‐term therapeutic option for patients in the end stage of chronic heart failure (CHF).

LVADs have been shown to significantly improve hemodynamics, mortality rates, and quality of life^1^, ^2^ but come with heightened risks for complications such as stroke, gastrointestinal bleeding, and device infection.^3^

Approximately 40% of patients with HF^4^ present clinically manifest cognitive dysfunction, ranging from mild cognitive impairment (MCI) to vascular dementia.^5^, ^6^

The underlying mechanisms are still being discussed, but the main causes seem to be associated with systemic inflammation and the reduction in cardiac output^5^, ^7^, ^8^ leading to chronic hypoperfusion of end organs and impaired organ function.

In the brain, this unphysiological environment provokes vascular and neuronal damage causing cerebral small vessel disease and cerebral microbleeds as well as neuroinflammation.^5^, ^7^, ^8^, ^9^

There is evidence for stabilization or improvement of cognitive capabilities following LVAD implantation, especially in the first year.^10^, ^11^, ^12^, ^13^, ^14^, ^15^, ^16^ However, some studies report postoperative cognitive decline in patients with LVAD.^13^, ^17^

Strokes seem to be the primary cause of cognitive impairment despite technologically improved devices.^13^, ^17^ Yet, previous studies identified structural cerebral changes such as a high burden of cerebral microbleeds and brain atrophy^18^, ^19^, ^20^, ^21^ as well as a variety of factors altering brain physiology, attributed to LVAD therapy. These are so far of rather hypothetical character, but include endothelial dysfunction, permanently increased sympathetic activation, abnormal cerebral autoregulation, diastolic hypertension, and a lack of cerebral clearance potentially leading to accumulation of β‐amyloid.^9^, ^22^, ^23^

^18^F‐fluorodeoxyglucose‐positron emission tomography/computed tomography (^18^F‐FDG‐PET/CT) of the brain is a well‐studied noninvasive molecular imaging technique of global and regional cerebral glucose metabolism, enabling early diagnosis of neurodegenerative disorders such as Alzheimer disease (AD) and frontotemporal dementia.^24^ In patients with LVAD, whole‐body ^18^F‐FDG‐PET/CT has become a valuable tool for assessing device‐related infection.^25^, ^26^, ^27^, ^28^ However, studies characterizing cerebral glucose metabolism using ^18^F‐FDG‐PET/CT in patients with LVAD are still lacking.

In this exploratory study, we aimed to assess neurocognitive performance of patients under long‐term LVAD therapy and patients with conservatively treated CHF under best medical treatment and for the first time relate the results to (1) serum biomarkers of neuroaxonal, and astrocytic activation as well as neurodegenerative markers NfL (neurofilament light chain protein), pTau181 (phosphorylated Tau protein at threonine 181), and GFAP (glial fibrillary acidic protein) and (2) cerebral glucose metabolism, quantified by ^18^F‐FDG‐PET/CT.

## METHODS

The data that support the findings of this study are available from the corresponding author upon reasonable request.

### Study Group and Clinical Data

Between September 2023 and May 2024, 32 patients with HF treated at the Department of Heart, Thoracic, Transplantation and Vascular Surgery or the Department of Cardiology at Hannover Medical School were prospectively enrolled into this exploratory cohort study. Nineteen patients were with and 13 without LVAD therapy. Patients were included when having an age over 18 years and a left ventricular ejection fraction <35%. We excluded all patients suffering from a malignant disease, a chronic disease of the central nervous system (eg a history of MCI or dementia), or a current sepsis according to the in‐house standard operating procedure^29^ to eliminate competing causes for cognitive impairment respectively cerebrometabolic alterations. Because the HeartWare HVAD System (HVAD, Medtronic) is known to be far less hemocompatible than the Heart Mate 3 (HM3, Abbott),^30^ we included only patients with the latter device and excluded all patients with HVAD accordingly. Due to the exploratory nature of the study and the lack of comparable data in the literature, a formal sample size calculation was not feasible; the university's institute for biometrics was involved during protocol development. A head imaging‐confirmed history of stroke was no exclusion criterion as strokes are a potential complication of LVAD therapy,^31^, ^32^, ^33^, ^34^ and therefore excluding these patients would present a selection bias. However, data analyses were done with and without exclusion of patients with former stroke.

Clinical characteristics including the length of LVAD support, the underlying heart disease, cardiovascular risk factors, routine laboratory parameters, and concomitant complications such as current infections were recorded (Data S1). Neurological status and functional capacity were evaluated using the National Institutes of Health Stroke Scale and modified Rankin Scale.

As screening instrument for MCI respectively dementia the Montreal Cognitive Assessment^35^, ^36^ was used to evaluate cognitive function. Based on the previous study by Yun and colleagues^37^ suggesting a decrease in frontotemporal metabolism in more severely advanced heart failure patients, we additionally assessed the Frontal Assessment Battery^38^ in all patients to evaluate frontal lobe dysfunction. Further information on both tests is attached in Data S2.

The study participants were screened for depression by a trained psychologist using the *Structured Clinical Interview for Diagnostic and Statistical Manual of Mental Disorders, 5th edition* and the Hospital Anxiety and Depression Scale.^39^

All patients underwent whole‐body static ^18^F‐FDG‐PET/CT to assess device infection or to screen for malignant diseases as part of heart transplantation evaluation. One patient with CHF received only myocardial ^18^F‐FDG‐PET/CT instead of a whole‐body scan and therefore could not be included in the ^18^F‐FDG‐PET/CT analyses of the brain, but for further analyses. Of note, this patient was 1 of the 2 patients with CHF with a history of stroke.

Opening characteristics of the aortic valve (AV) during contraction were retrospectively acquired from the latest echocardiography before study inclusion. Each patient was categorized in 1 of the following groups: (1) AV opens actively with (almost) every contraction, (2) AV opens occasionally, and (3) AV opens just minimally or not at all. In 4 patients the AV could not be visualized on echocardiography.

The study protocol complied with the Declaration of Helsinki and was approved by the institutional review board at Hannover Medical School (No. 10790_BO_K2023). All patients gave written informed consent allowing the use of medical data for scientific purposes and blood withdrawal.

### PET/CT Imaging and Image Analysis

Thirty‐one patients underwent whole‐body static ^18^F‐FDG PET/CT using a Biograph mCT Flow 128 scanner (Siemens Healthineers, Erlangen, Germany).

Statistical parametric mapping (SPM8) implemented in Matlab Version 8.4.0.150421 (R2014b) (The Mathworks Inc., Natick, MA, USA) was applied for image processing and analysis. A healthy control group is mandatory in the SPM analyses. We therefore retrospectively selected 16 subjects without cardiac and neurological disease including 5 men and 11 women (median age 62 years [49–66]) who underwent ^18^F‐FDG‐PET/CT with unremarkable findings using the same imaging protocol and the same scanner as the patients with HF.

The detailed procedure is enclosed in the supplements as well as further information on the workflow of ^18^F‐FDG‐PET/CT acquisition, data preprocessing, and image analysis in order to obtain voxel values for all hypometabolic clusters (Data S3).

Image analysis was performed using (1) single subject versus control group (SSVCG) analyses to detect metabolic alterations, that is, hypo‐ and hypermetabolic clusters of cohesive voxels in each patient individually, and (2) SPM's 2‐sample *t* test for group comparisons to determine differences between the different patient groups. A *P* value <0.001 uncorrected for multiple testing was applied.

Figure 1 illustrates the SSVCG results of 2 patients with LVAD as examples—1 with (A) and 1 without (B) a history of stroke.

**Figure 1:**
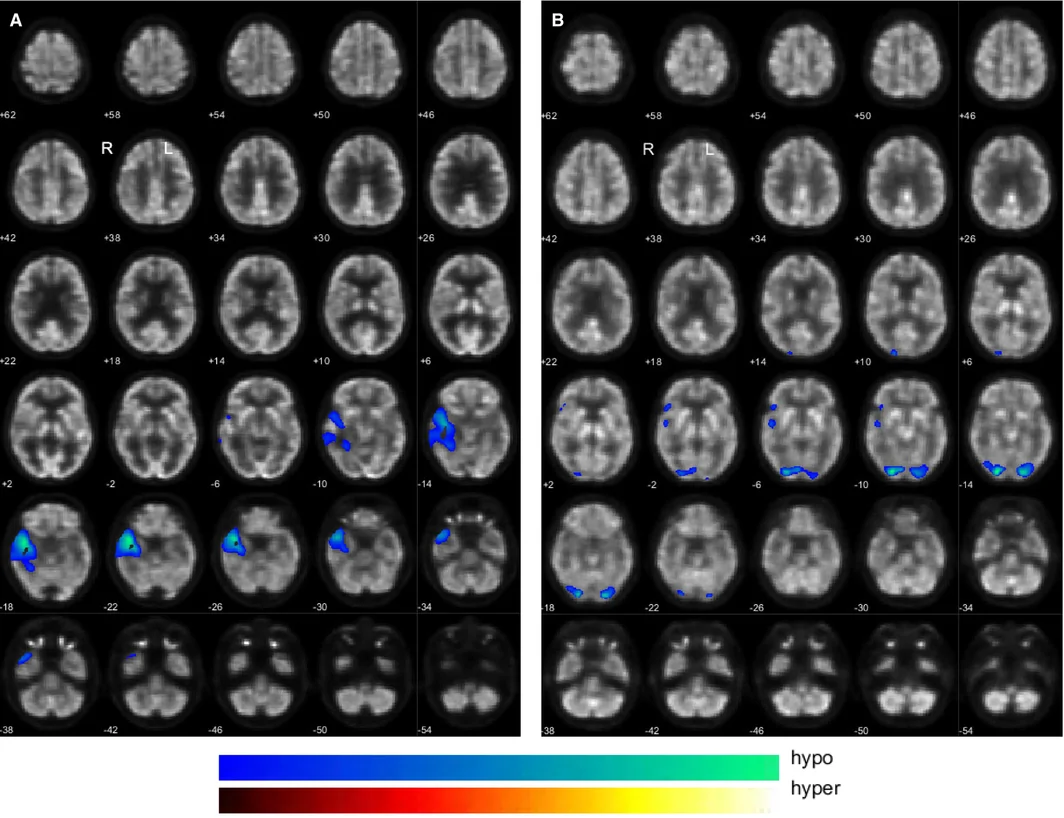
Combined SPM results for hypo‐ and hypermetabolic contrast exemplarily from 2 of our 31 patients with heart failure who received whole‐body ^18^F‐FDG‐PET imaging (*P*<0.001 uncorrected). **A**, Shows primarily a hypometabolic cluster (volume: 34.96 mL) in the right temporal lobe consistent with the location of a suffered stroke. **B**, Shows several hypometabolic clusters in both occipital lobes and the right temporal lobe in an LVAD patient without a history of stroke. ^18^F‐FDG‐PET/CT indicates 18F‐fluordeoxyglucose positron emission tomography/computed tomography; LVAD, left ventricular assist device; and SPM, single‐subject statistical parametric mapping.

### Biomarker Analysis

Blood samples were obtained from patients with LVAD and CHF on the same day as ^18^F‐FDG‐PET/CT acquisition, processed according to standardized procedures, and stored at −80 °C until analysis. Serum biomarkers were quantified with commercially purchased immunoassays, namely the Simoa GFAP Discovery Kit and the pTau‐181 Advantage PLUS kit run on a HD‐X platform (Quanterix Inc., Lexington, USA) for GFAP and pTau181, respectively, and the Human NF‐L Simple Plex assay for the Ella microfluidic system (BioTechne, Minneapolis, MN, USA) for NfL. Internal controls were run to calculate intra‐ and interassay coefficients of variability, which were <15% and <20%, respectively.

### Statistical Analysis

Statistical analyses were performed using IBM SPSS‐Statistics for Windows (v.29.0.1.0; IBM‐Deutschland) and R studio (v.4.5.1; R Foundation, Vienna, Austria). R studio and SPM were also used to visually present data when necessary. Graphical abstract was created with BioRender.com. Categorical variables are reported in frequencies and continuous variables as median with interquartile range (IQR). In univariate analysis of baseline characteristics, the Pearson chi‐square test was applied for categorical variables. Group differences in continuous variables were evaluated using Mann–Whitney test for independent samples. CIs were calculated using Hodges–Lehmann estimator. Correlation analysis was performed with the Spearman's rank correlation coefficient to identify similarities between neurodegenerative outcome parameters (cerebral hypometabolic volume, neurocognitive test results, biomarker assessments) and clinical measurements. The Kruskal–Wallis test was used to compare the categories of AV opening on the outcome parameters. Linear regression models were conducted to adjust the outcome variables—in deference of the small sample size of n=32 limited to 3 variables—for the most significant confounders (ie, age, arterial hypertension, history of stroke).

The Benjamini–Hochberg method was applied to adjust the *P* value in order to reduce the bias caused by multiple tests. The regression models are likely overfitted and lack statistical robustness due to the low ratio of event to variables, and their results should therefore be considered with caution. Statistical significance was defined as a *P* value of <0.05.

## RESULTS

We screened 40 patients with advanced HF who were scheduled for ^18^F‐FDG‐PET/CT, of whom 32 were recruited (2 patients suffered from a sepsis at time of ^18^F‐FDG‐PET/CT, 3 were implanted with a HVAD, and 3 declined study inclusion). Demographics, clinical characteristics, and neurocognitive/biomarker assessment results are presented in Table 1. Prescribed medication potentially affecting glucose metabolism according to the European Association of Nuclear Medicine procedure guidelines for brain PET imaging are attached in Table S3.^24^

**Table 1:** Baseline Characteristics at Time of ^18^F‐FDG‐PET/CT Compared Between Patients With LVAD and CHF

Parameters	LVAD	No.	CHF	No.	*P* value
Age, y	59 (52–66)	19	47 (40–64)	13	0.147
Sex, male	16 (84.2%)	19	9 (69.2%)	13	0.314
Education, y	13 (13–15)	19	13 (12.5–14.5)	13	0.734
Cardiovascular risk factors					
Arterial hypertension	13 (68.4%)	19	3 (23.1%)	13	0.012*
Mean arterial pressure (at time of PET/CT in mm Hg)	80 (70–94)	19	79.5 (74.8–95.5)	13	0.821
Diabetes	6 (31.6%)	19	2 (15.4%)	13	0.299
Hyperlipidemia	8 (42.1%)	19	3 (23.1%)	13	0.266
Body mass index, kg/m^2^	27.8 (25.2–35)	19	26.6 (20.9–28.5)	13	0.092
History of smoking	16 (84.2%)	19	5 (38.5%)	13	0.007*
Pack years	20 (10–45)	19	0 (0–15)	13	0.003*
Cardiomyopathy		19		13	0.243
Dilated cardiomyopathy	8 (42.1%)		6 (46.2%)		
Ischemic cardiomyopathy	10 (52.6%)		4 (30.8%)		
Others	1 (5.3%)		3 (23.1%)		
New York Heart Association class		19		13	0.835
1	0 (0%)		0 (0%)		
2	5 (26.3%)		2 (15.4%)		
3	14 (73.7%)		11 (84.6%)		
4	0 (0%)		0 (0%)		
Atrial fibrillation	6 (31.6%)	19	6 (46.2%)	13	0.403
CHA2DS2‐VASc score	5 (4–6)	19	3 (3–3)	13	0.061
Stroke	9 (47.4%)	19	2 (15.4%)	13	0.061
Modified Rankin Scale score	3 (1–3)	19	2 (1–3)	13	0.343
National Institutes of Health Stroke Scale score	1 (0–2)	19	0 (0–0.5)	13	0.147
MoCA score	25 (23–26)	19	25 (21.5–26.5)	13	0.85
MoCA severity category	1 (0–1)	19	1 (0–1)	13	0.91
No cognitive impairment (≥26)	6 (31.6%)		4 (30.8%)		
Mild cognitive impairment (19–25)	12 (63.2%)		8 (61.5%)		
Moderate cognitive impairment (11–18)	1 (5%)		0 (0%)		
Severe cognitive impairment (10)	0 (0%)		1 (8%)		
Frontal Assessment Battery score	16 (14–17)	19	17 (16–18)	13	0.383
Major depressive disorder (at time of PET/CT)	4 (28.6%)	16	5 (38.5%)	11	0.383
Laboratory values (at time of PET/CT)					
C‐reactive protein, mg/L	8.1 (4.9–18.5)	19	5.2 (1.8–11.9)	13	0.182
White blood cells, 10^3^/μL	6.8 (6.1–8)	19	6.7 (5.2–8.8)	13	0.910
N‐terminal pro–brain natriuretic peptide, last available, ng/L	935 (544–2042)	19	4882 (1702–14 220.5)	13	0.006*
Estimated glomerular filtration rate (Chronic Kidney Disease Epidemiology Collaboration, mL/min per 1.73 m^2^)	51 (40–70)	19	47 (24–57.5)	13	0.448
Creatine, μmol/L	126 (104–157)	19	137 (108.5–248.5)	13	0.623
^18^F‐FDG‐PET/CT					
Megabecquerel	262 (231–326)	19	222 (177.3–257.8)	12	0.039*
S‐glucose (at time of PET/CT, mmol/L)	5.1 (4.6–5.9)	19	5.5 (4.1–6.7)	12	0.734
Total hypometabolic volume (mL)	1.03 (0–7.34)	19	0 (0–0.01)	12	0.011*
LVAD‐specific characteristics					
Duration of LVAD support, d	517 (217–1170)	19			
Device infection (infected components: driveline entry point, subcutaneous driveline path, pump pocket, outflow tract)		19			
1	6 (31.6%)				
2	6 (31.6%)				
3	4 (21.1%)				
4	3 (15.8%)				
Implant technique (minimally invasive)	4 (21.1%)				
Aortic valve opening during the ejection phase		19			
(Almost) always	6 (31.6%)				
At times	3 (15.8%)				
Never/seldom	6 (31.6%)				
Could not be visualized	4 (21.1%)				

Results are displayed as frequencies (percentages) and metric data as median with interquartile range (25th–75th percentile). ^18^F‐FDG‐PET/CT indicates ^18^F‐fluordeoxyglucose positron emission tomography/computed tomography; CHF, chronic heart failure; LVAD, left ventricular assist device; and MoCA, Montreal Cognitive Assessment.

* *P* values <0.05 are considered statistically significant.

Patients with LVAD were in median 59 (IQR, 52–66) and patients with CHF 47 (40–64) years old. Sixteen (84.2%) patients with LVAD and 9 (69.2%) patients with CHF were male, 3 (15.8%) patients with LVAD and 4 (30.8%) patients with CHF were female. The median years of education were 13 years in both groups (IQR_LVAD_, 13–15; IQR_CHF_, 12.5–14.5). Underlying cause of cardiomyopathy in patients with LVAD was ischemic cardiomyopathy (n=10), followed by dilated cardiomyopathy (n=8) and valvular genesis in 1 patient. Four patients with CHF had ischemic cardiomyopathy, 6 dilated cardiomyopathy, and 3 other causes (ie, congenital heart disease, postmyocarditis).

Patients with LVAD had significantly more cardiovascular risk factors: more frequently arterial hypertension (LVAD: n=13, CHF: n=3, *P*=0.012), more frequently a history of smoking (LVAD: n=16, CHF: n=5, *P*=0.007) and more pack years (LVAD: 20 (10–45), CHF: 0 (IQR, 0–15), *P*=0.003). A trend could be seen regarding a higher body mass index (LVAD: 27.8 [IQR, 25.2–35], CHF: 26.6 [IQR, 200.9–28.5], *P*=0.092).

Patient groups did not differ regarding other risk factors for cognitive impairment: history of stroke, blood parameters of inflammation, renal and hepatic function, and prevalence of depression at the time of ^18^F‐FDG‐PET/CT.

Of note, the patients with LVAD were about 8 years older than the patients with CHF and presented a greater cardiovascular burden.

All patients with LVAD were implanted with centrifugal‐flow pump HM3. The duration of LVAD support at the time of the ^18^F‐FDG‐PET/CT was in median 517 days (IQR, 217–1170).

Out of the 19 HM3 patients, 6 (31.6%) patients had an AV opening at (almost) every contraction, and 3 (15.8%) patients had their AV opening occasionally during the ejection phase. The AV of another 6 (31.6%) patients opened never or just seldom, and 4 (21.1%) patients could not be categorized. Kruskal–Wallis test did not show a significant difference for any of our neurological outcome parameters.

Nine patients (LVAD n=4, CHF n=5) were diagnosed with depression at time of ^18^F‐FDG‐PET/CT according to the *Structured Clinical Interview for Diagnostic and Statistical Manual of Mental Disorders, 5th edition*, of whom 3 patients were under treatment with antidepressant medication.

### Neurocognitive Functions

Median Montreal Cognitive Assessment scores were 25 points (IQR, 23–26) in the LVAD group and 25 points (IQR, 21.5–26.5) in the group with CHF, with 68.4% (LVAD group) and 69.5% (group with CHF) of the patients having a Montreal Cognitive Assessment score <26 (ie, MCI). The MCI severity category ranges were spread similarly in both groups (*P*=0.91), which is reflected by the group comparison (*P*=0.842 [95% CI, −2 to 2]) (Figure 2A). The results of patients with a history of stroke did not differ from those without (*P*=0.714 [95% CI, −3 to 2]) (Figure 2B).

**Figure 2:**
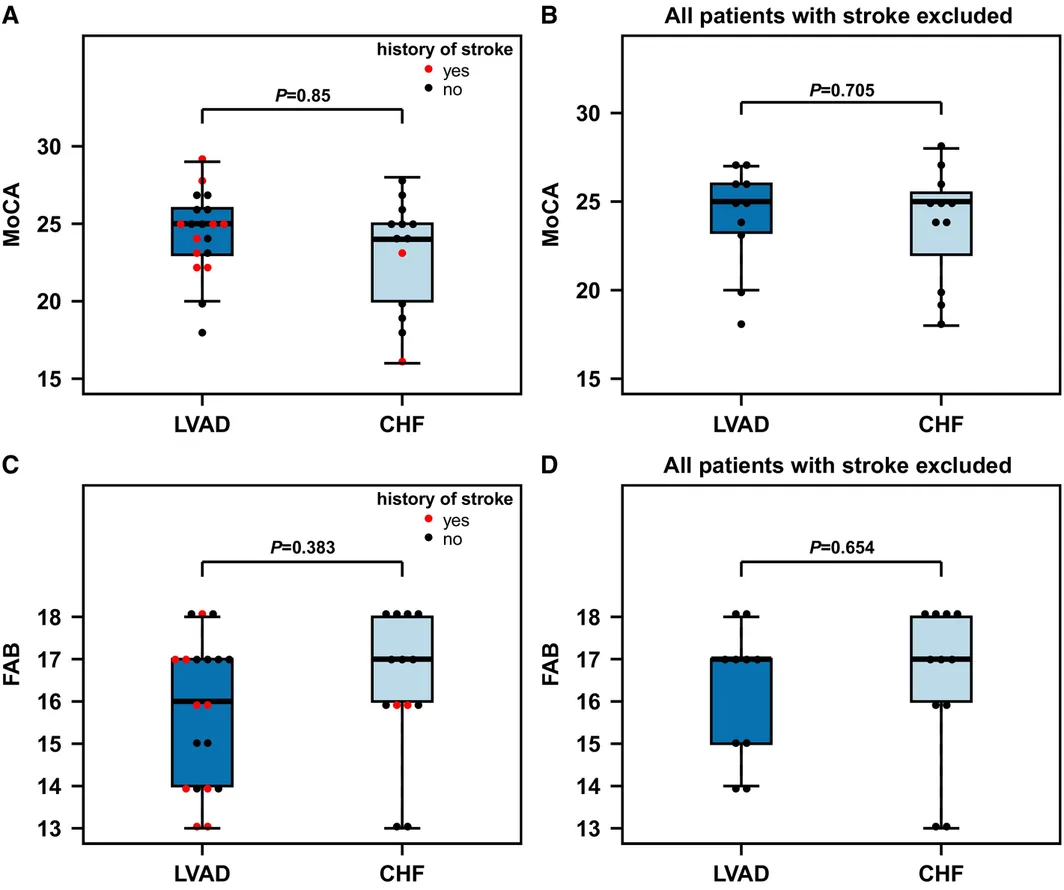
Boxplots of cognitive test results in patients with LVAD and CHF. **A**, Montreal Cognitive Assessment scores show impaired cognitive performance based on a median MoCA result of 25 of 30 points in both groups. **B**, All patients with a history of stroke were excluded from both groups (9 with LVAD and 2 with CHF), still revealing impaired cognitive function. **C**, Frontal Assessment Battery scores in patients with LVAD and CHF. **D**, All patients with a history of stroke were excluded from both groups (9 LVAD and 2 CHF). CHF indicates chronic heart failure; FAB, Frontal Assessment Battery; LVAD, left ventricular assist device; and MoCA, Montreal Cognitive Assessment.

The Frontal Assessment Battery results revealed frontotemporal impairment in 5 patients: n=3 with LVAD and n=2 with CHF. The 2 groups did not show significant differences in the Frontal Assessment Battery results (*P*=0.368 [95% CI, −1 to 2]) (Figure 2C), even after excluding all patients with a history of stroke (*P*=0.637 [95% CI, −1 to 2]) (Figure 2D) (3 of the 10 patients with a history of stroke had a residing infarct core in the frontal lobe).

The most frequently impaired cognitive functions in our patient groups were concordant tasks in the cognitive assessment for short‐ and long‐term memory retrieval, verbal fluency, and attention (see Figure 3).

**Figure 3:**
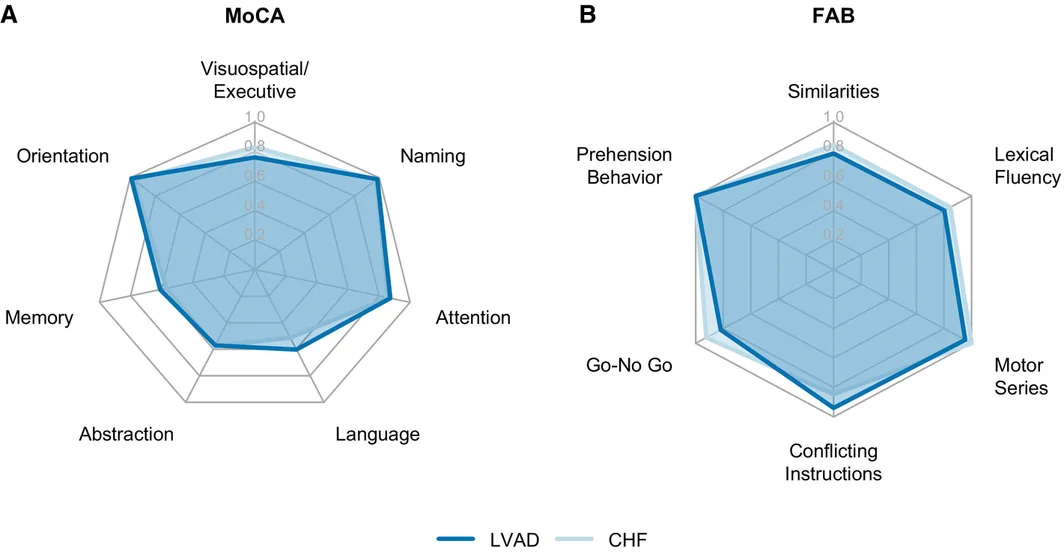
Radar charts of the cognitive tests' exercise domains of patients with LVAD and CHF. The mean percentage of right results in both patient groups are listed on the radial axis separated for all subdomains. **A**, MoCA subdomains show similar results for both groups. The most prominently impaired categories were visuospatial/executive, memory, abstraction, and language. **B**, FAB tasks show similar results for both groups. The most prominently categories were similarities, lexical fluency and the Go‐No Go task. CHF indicates chronic heart failure; FAB, Frontal Assessment Battery; LVAD, left ventricular assist device; and MoCA, Montreal Cognitive Assessment.

### Cerebral Glucose Metabolism

The SPM group comparison of patients with LVAD and CHF against the 16 control subjects revealed a frontotemporally accentuated cerebral hypometabolism in both patients with LVAD and CHF (*P*<0.001 uncorr.) (Figure 4A and 4C). The frontotemporal hypometabolism was less pronounced in patients with CHF (Figure 4C) than in the patients with LVAD (Figure 4A).

**Figure 4:**
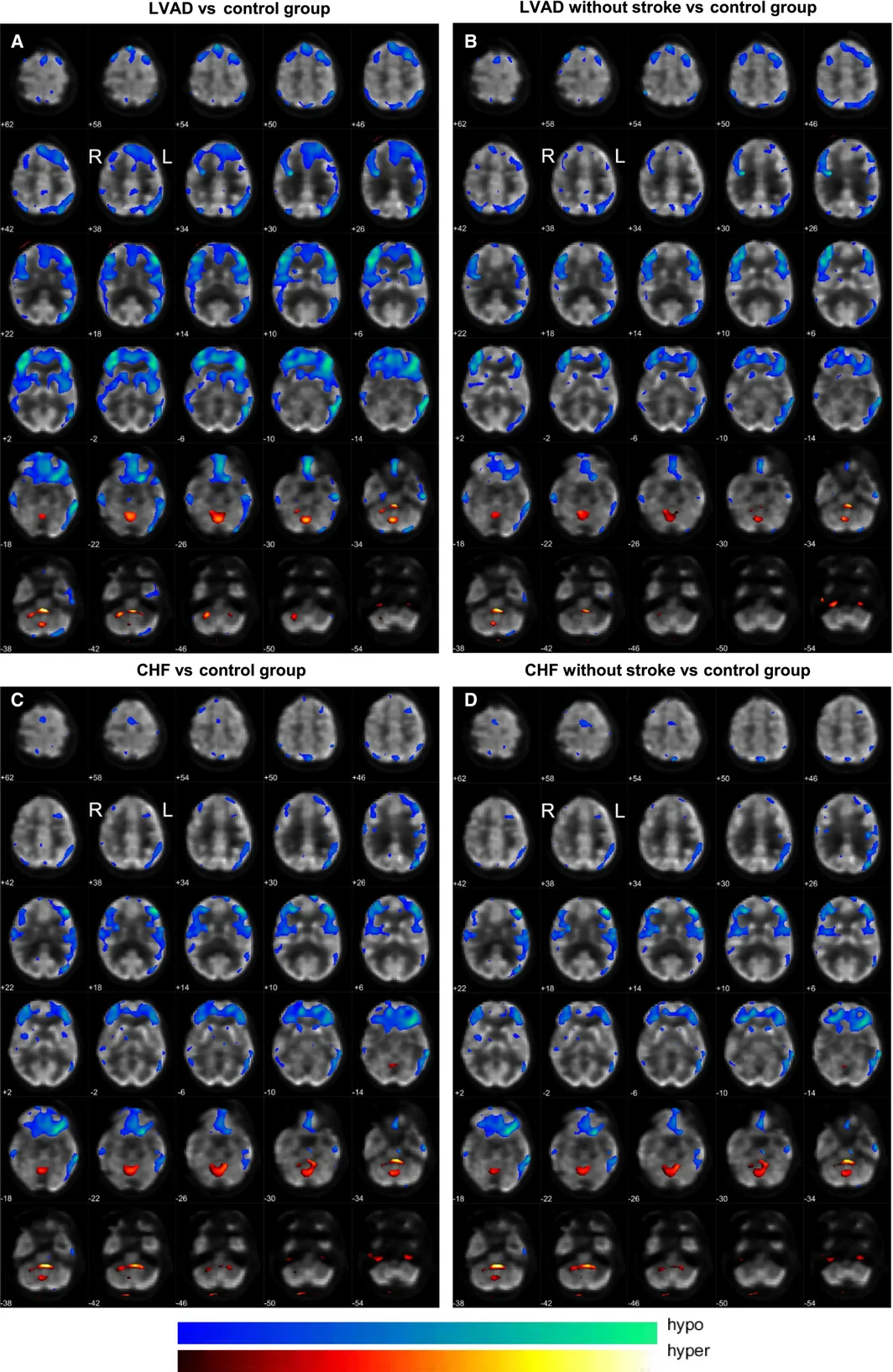
Combined results of group comparisons of all patients for suprathreshold hypo‐ and hypermetabolism of SPM 2‐sample *t* test (*P*<0.001 uncorrected) in ^18^F‐FDG‐PET/CT visualized as sectional images. **A**, All 19 patients with LVAD tested against the 16 healthy control subjects revealed a frontotemporally accentuated cerebral hypometabolism. **B**, Exclusion of all patients with a history of stroke (n=9) decreased extent of significant hypometabolism but proved the frontal pattern. **C**, All 13 patients with CHF tested against the 16 healthy control subjects revealed a comparable, but less pronounced frontotemporally accentuated hypometabolism. **D**, The exclusion of all patients with a history of stroke (n=2) revealed a similar result. ^18^F‐FDG‐PET/CT indicates ^18^F‐fluordeoxyglucose positron emission tomography/computed tomography; AD, Alzheimer's disease; CHF, chronic heart failure; LVAD, left ventricular assist device; and SPM, single‐subject statistical parametric mapping.

The most distinct hypometabolic regions in both patient groups were located in frontal and temporal lobe bilaterally with left‐hemispheric pronounced extensions in the parietal lobe. Occipital lobe, cerebellum, and brainstem were mostly spared. The exclusion of all patients with a history of stroke decreased the extent of significant hypometabolism but proved the frontal pattern (Figure 4B and 4D). It should be noted that the imaging results rely on significance thresholds uncorrected for multiple testing, while diminishing under standard correction (*P*<0.05 family‐wise error [FWE] corrected). The FWE correction, however, produced verifiably false‐negative results in SSVCG analyses (for further details see Data S1).

The LVAD group revealed cerebral metabolic alterations with a median total hypometabolic volume (THV) of 1.03 mL (IQR, 0–7.34 mL), which was significantly greater than in the group with CHF (0 mL, IQR, 0–0.1 mL; *P*=0.011 [95% CI, −6.128 to 0]) (Figure 5A). The difference barely misses significance after excluding all patients with a history of stroke (LVAD: 0.69 mL [0–5.28], CHF: 0 mL [0–0]; *P*=0.051 [95% CI, −1.576 to 0]), though stroke lesions produce partially large hypometabolic clusters (Figure 5B).

**Figure 5:**
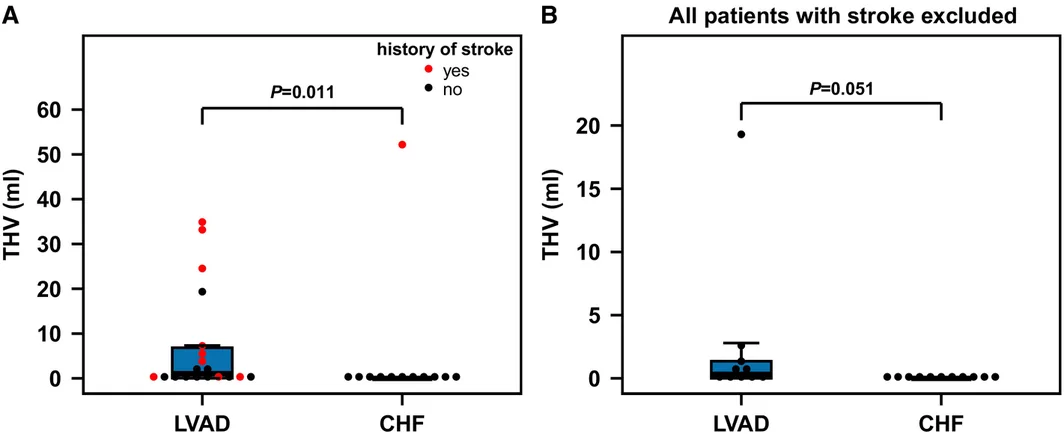
Boxplots of the total hypometabolic volume measured by ^18^F‐FDG‐PET/CT in (A) patients with LVAD and CHF showing significantly greater hypometabolism in patients with LVAD (*P*<0.05) and (B) All patients with a history of stroke were excluded from both groups (9 with LVAD and 1 with CHF). The results are more likely to be attributed to the substantial differences in age and cardiovascular risk factors than device therapy. ^18^F‐FDG‐PET/CT indicates ^18^F‐fluordeoxyglucose positron emission tomography/computed tomography; CHF, chronic heart failure; LVAD, left ventricular assist device; and THV, total hypometabolic volume.

No significant effect of age on THV could be seen in the whole LVAD group (Spearman's ρ=0.141 [95% CI, −0.347 to 0.569], *P*=0.564) nor the group with CHF (ρ=0.138 [95% CI, −0.488 to 0.67], *P*=0.669). However, excluding all patients with a history of stroke revealed a significant association between age and THV in patients with LVAD (ρ=0.634 [95% CI, −0.015 to 0.907], *P*=0.049). The correlation analyses lack statistical power due to the small sample size. The significant difference in THV is more likely to be attributed to the substantial differences in age and cardiovascular risk factors than device therapy.

An association between the cognition test results and the THV could not be reported.

### Blood‐Based Biomarkers

Serum samples were available from all 32 patients with HF, with just 1 missing value of a LVAD patient in the GFAP immunoassay.

Serum NfL, pTau181 and GFAP levels were elevated compared with previously reported healthy and controls and controls with HF^40^, ^41^, ^42^, ^43^, ^44^, ^45^, ^46^, ^47^, ^48^ (Table S1) but none of the 3 biomarkers differed significantly between patients with LVAD and CHF (Table 2), even after excluding all patients with a history of stroke (Figure 6). Associations of impaired brain metabolism, serum biomarkers of neuronal injury/activation, and cognitive performance are attached in Data S1.

**Table 2:** Comparison of Serum Concentrations of Neuronal Biomarkers NfL, pTau181, and GFAP Between Patients With LVAD and CHF

	LVAD			CHF			*P* value (95% CI)
	Median (IQR)	Range	n	Median (IQR)	Range	n	
NfL, pg/mL	44.5 (32–79)	8.52–384.5	19	48.4 (17.2–96)	4–339	13	0.91 (95% CI, −30.8 to 36.75)
pTau181, pg/mL	22.1 (19.5–34.5)	15.4–63.5	19	25.7 (17.2–67.8)	13.7–84.2	13	0.57 (95% CI, −4.6 to 20.9)
GFAP, pg/mL	194.8 (126–299)	83.9–365.8	18	190.1 (130–231.2)	65–617.7	13	0.77 (95% CI, −77.4 to 63.8)

Serum concentrations are quoted in pg/mL as median with interquartile range (25th–75th percentile) and overall range (minimum‐maximum). *P* values <0.05 is considered statistically significant. CHF indicates chronic heart failure; GFAP, glial fibrillary acidic protein; IQR, interquartile range; LVAD, left ventricular assist device; NfL, neurofilament light chain protein; and pTau181, phosphorylated Tau protein at threonine 181.

**Figure 6:**
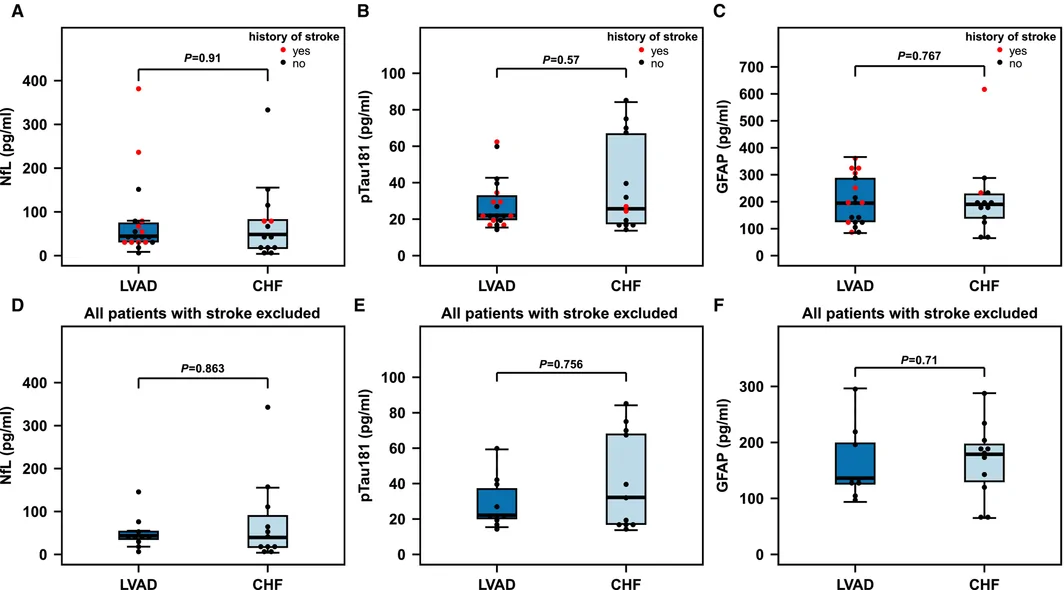
Boxplots of serum concentrations of biomarkers of cerebral injury in patients with LVAD and CHF. **A** NfL, **B** pTau181, and **C** GFAP all showed elevated levels compared with healthy controls (see Table S2). **D, E**, **F,** All patients with a history of stroke were excluded from both groups (9 with LVAD and 2 with CHF) revealing similar results. CHF indicates chronic heart failure; GFAP, glial acidic fibrillary protein; LVAD, left ventricular assist device; NfL, neurofilament light chain protein; and pTau181, tau protein phosphorylated at threonine 181.

### Regression Models

Linear regression models were tested for each outcome variable adjusted for the most significant confounders: age, arterial hypertension, and history of stroke. Following the small sample size, they were most likely underpowered.

The only significant result was an independent effect of history of stroke on the THV in the whole cohort with HF (corrected R^2^=0.274, β=14.86 [95% CI, 5.796–23.93], *P*=0.002). All other regression models missed the significance threshold after correction for multiple testing.

## DISCUSSION

With the improvement in survival rates of patients with HF after LVAD implantation and the introduction of the HM3 device, the importance of long‐term neurocognitive outcomes^49^ has increased.

We conducted this prospective exploratory study of 32 patients with severe HF—19 with LVAD and 13 without—to further investigate the cerebral impairment in patients with HF after LVAD implantation by (1) cognitive testing, (2) cerebral glucose metabolism measured by ^18^F‐FDG‐PET/CT, and (3) serum biomarkers of neuronal and astrocytic injury.

Few previous studies investigated cerebral metabolic changes using ^18^F‐FDG‐PET^6^, ^37^, ^50^, ^51^ or neuronal/astrocytic serum biomarkers^44^, ^45^, ^46^, ^47^ in patients with HF. To our knowledge, studies on neurocognitive outcome in patients with LVAD have been limited to cognitive testing; thus we present the first study that investigated cerebral metabolism and serum neuronal and astroglial markers in patients with LVAD.

The main findings of our investigation were (1) diminished cognitive capabilities in patients with severe HF, (2) elevated serum biomarker levels and (3) indications of a frontotemporally accentuated cerebral hypometabolism independent of a history of stroke in both groups of patients with severe HF—namely in those with and those without mechanical circulatory support.

### Cognitive Performance

The most frequently impaired cognitive functions in our patient groups—short‐ and long‐term memory retrieval, verbal fluency, and attention (Figure 3)—are related to the affected cerebral regions seen in the group comparisons, that is, merely frontal and temporal lobe (Figure 4). These findings correspond to recent studies that found cerebral metabolic impairments in concordant frontal^51^, ^52^ and temporal^6^, ^50^, ^51^, ^52^, ^53^ cognition‐related regions in patients with HF. These regions are assumed to be primarily affected by hypometabolism due to a high susceptibility to hypoperfusion and hypoxia^50^, ^52^, ^53^ or as a possible correlate of concomitant depressive symptoms.^51^

However, an association between FDG metabolism and cognitive performance should be regarded with caution for several reasons: (1) the snapshot character of the ^18^F‐FDG‐PET/CT thus the inability to display longitudinal dynamics, (2) the known initial recovery after LVAD implantation^10^, ^11^, ^12^, ^13^, ^14^, ^15^, ^16^ (3) reduction in cerebral metabolism that may precede onset of clinical symptoms by years,^54^ and (4) variable cognitive reserve dependent on individual factors and the overall comorbidity burden.^55^ Moreover, alterations of FDG metabolism with concomitant brain atrophy patterns^37^ only partially overlap with elevated blood biomarker levels.^56^ For instance, this is seen depending on the underlying disease in frontal areas (eg, NfL) and hippocampus (eg, pTau181). Hence, fluid and nuclear imaging markers may rather reflect patients with HF and LVAD and be used as complementary tools in future validation studies.

### Cerebral Glucose Hypometabolism

At first, when interpreting the PET‐findings, it should be considered that using an uncorrected *P* value without multiplicity correction carries the high risk of false positive results. Therefore, positive, that is, hypometabolic, findings should not be overstated but rather considered alongside the other outcome variables.

A *P* value <0.05 with FWE correction presents a more rigorous threshold ideally reducing the risk for false positives, potentially explaining the extensive reduction of detected significant hypometabolism in our results. However, false negative results after FWE correction cannot be excluded either, indicated for example by the missing detection of a magnetic resonance imaging‐validated infarct core (Figures S1 and S2; Table S2). Further verification was not feasible missing dynamic data. Thus, and in regard of the study's exploratory approach, we decided on applying the threshold with lesser evidence for false results.

The cerebral metabolism is known to decrease with age.^57^, ^58^ Although not statistically significant, the patients with LVAD included in this study were in median around 12 years older and presented with higher prevalence of cardiovascular risk factors.

Due to the shortage of donor organs, heart transplantation is preferably offered patients with good physical constitution facilitating survival of the transplantation surgery and following immunosuppression. This is often accompanied by a younger chronological age and a lower cardiovascular burden. However, these patients with HF were the only ones regularly receiving whole‐body FDG‐PET imaging.

The greater baseline vascular burden in the patients with LVAD not only contributes to higher prevalence of cerebral strokes. It also leads in smaller scale to cerebral small vessel disease as well as microvascular hypoperfusion and consequently neuronal damage^5^, ^7^, ^8^, ^9^ and hypometabolism.^51^ Therefore, the greater THV measured in the LVAD group might be partially based on the worse state of cerebral perfusion following the greater vascular burden.

Moreover, it should be discussed whether more severely affected patients with CHF with left ventricular ejection fraction <25% could serve as a more compatible control group when tolerating the ^18^F‐FDG‐PET/CT examination, especially because an effect of the left ventricular ejection fraction on brain metabolism has been demonstrated in the past.^37^, ^59^

Our data present a considerable discrepancy between the SSVCG analyses that detect—apart from larger infarct cores—discreet hypometabolic defects opposing to the more widespread reduction in brain metabolism, particularly frontotemporally, seen in the group comparisons. We hypothesize that the metabolic derogation affecting most of the cerebral gray matter areas due to cerebral microangiopathy and thus hypoperfusion is too subtle to be detected in SSVCG analyses, which is why SPM group comparisons are needed to raise the frontotemporal hypometabolism to detectability. The apparent difference there between the groups with LVAD and CHF might be at least partially attributed to the greater vascular burden in our patients with LVAD, as discussed here.

Our small sample size and disparate cardiovascular risk profiles prevent data‐based interpretations on potential device‐related effects on cerebral functionality. However, justifiable questions remain because patients with LVAD are vulnerable to both bleeding and thrombotic events. The influence of duration of device support, the device type itself and the status of remaining AV opening capabilities still remains poorly investigated.

Of the 2 dominantly implanted devices in recent years, the centrifugal flow HVAD system presented a higher risk of strokes compared with the HM3 device,^34^ consequently leading to its end of distribution in 2021.

Additionally, the HVAD is known to be less hemocompatible with higher levels of hemolysis compared with the HM3.^30^

Therefore, HVAD patients may have an increased susceptibility for subclinical brain injury (eg, cerebral microbleeds)—conceivably comparable to hemolytic anemia such as sickle‐cell disease^60^—that could cause over time manifest cognitive symptoms.^13^

The other centrifugal‐flow pump design HM3 aims for better hemocompatibility through magnetic levitation of the pump, wider blood‐flow pathways, and an artificial pulse unsynchronized to the heart rate^61^ and thus has a different effect on the vascular physiology.

A similar issue is the influence of AV opening characteristics. A permanently closed AV leads to stasis in the aortic root thus promoting the formation of aortic root thrombus, potentially increasing the risk for cerebral macro‐ and microangiopathy.^62^ However, we could not detect a significant effect of AV opening on any of our outcome parameters, which could be due to the underpowered analysis in our small‐size exploratory cohort.

### Blood‐Based Biomarkers

Likewise, to the cognitive performance, the neurochemical biomarker levels did not provide evidence for an additional detrimental effect of LVAD therapy on the central nervous system on top of the underlying chronic heart failure.

The serum NfL levels of our patients were elevated in comparison to reference data in the literature^40^, ^41^, ^42^, ^43^ (Table S1) indicating ongoing neuroaxonal damage. Strokes are a potential complication in patients with LVAD.^31^, ^32^, ^33^, ^34^ In the acute phase of stroke, the axonal intermediary filament NfL is released into the serum from the ischemic tissue up to weeks after stroke onset^63^ and declining thereafter over several months showing elevated levels at times for up to a year.^64^ The acute increase can be attributed to the initial neuronal death caused by the ischemic event. However, the chronically declining elevation reflects neuronal degeneration through Wallerian degeneration as well as protein turnover during the recovery period.^65^ Most of our patients experienced their stroke >1 year before study inclusion. It is hence possible that elevated NfL concentrations may reflect chronic neuronal damage in its multifactorial nature, being associated for example with chronic cerebral vascular burden due to continuous microbleeds and silent strokes,^66^, ^67^, ^68^ impaired cerebral perfusion, proinflammatory disposition, endothelial dysfunction, and neurohumoral dysregulation.^8^, ^46^, ^69^, ^70^

In comparison to recent studies investigating neuronal biomarkers in patients with HF, our patients had greater serum levels of NfL and pTau181, probably due to on average less severely impaired cardiac function in the formerly studied groups.^44^, ^45^, ^46^, ^47^ Instead, GFAP levels were of comparable magnitude, but a relevant cohort‐related bias cannot be excluded.

GFAP is a crucial component of the cytoskeleton of astrocytes. Its increased expression can indicate reactive astrogliosis due to neighboring neuronal death as well as blood–brain barrier disruption.^71^

For patients with HF, factors such as cerebral hypoperfusion, hypoxia, and neuroinflammation may contribute to this elevation.

The positive correlation of serum GFAP levels and THV in the patients with LVAD without a history of stroke supports this relation,^72^ although larger studies accounting for possible confounding factors^42^ are required to validate this hypothesis.

Plasma pTau181 is a well‐known biomarker of tau hyperphosphorylation, which has been associated with the earliest pathophysiological events of AD‐related neurodegeneration.^73^ Despite the valuable use as a core 1 AD biomarker according to the most updated criteria for diagnosis of AD,^73^ its specificity for AD is still controversial given its associations also with other neurodegenerative diseases such as amyotrophic lateral sclerosis.^74^ Hence, the interpretation of pTau181 levels in patients with CHF may be not univocal. On the one hand, the longitudinal increase of pTau181 levels over years may suggest the beginning of CHF‐related neurodegenerative processes,^45^ also in the context of AD‐like patterns. On the other hand, given the influence of comorbidities on pTau181 levels such as renal disease, it is possible that pTau181 levels in patients with CHF may not be strictly associated with incipient AD.

Additionally, hypometabolic regions characteristically affected in AD such as the posterior cingulate cortex, the precuneus, and the posterior temporoparietal cortex^75^ are significantly less affected and a predominantly frontotemporal impairment could be observed in our PET analyses. However, considering the new understanding of AD as wide continuum beginning years before clinical onset,^76^ our findings underline the importance of further investigations to unveil the pathophysiology of cognitive impairment in CHF.

## LIMITATIONS

Several limitations are to be considered when interpreting our study.

First, the small sample size limits the generalizability of our results. For the same reason, analyses on patient subgroups with testing for the extent of driveline infection, AV opening characteristics, or pathogenetic cardiovascular pathology for example were greatly hampered. Investigating sex‐specific differences was likewise limited to descriptive measurements.

The considerable difference in age and cardiovascular risk factors limits the comparability of our 2 groups as an age difference of about 12 years and a higher vascular burden between the groups with LVAD and CHF are potential confounders for brain metabolism, cognitive performance and biomarkers of cerebral injury. Due to the small sample size, sufficient multivariate models adjusting for potential confounders were limited to 3 independent variables.

Additionally, because the healthy control groups' age distribution matches the LVAD group reasonably well, the risk for false negative findings in the group with CHF is to be considered.

Hence, our findings should be interpreted as exploratory, hypothesis‐generating, and requiring extensive validation.

The cross‐sectional design lacks comparable pre‐LVAD implantation as well as longitudinal follow‐up data and thus misses information on dynamics of the measured parameters. Additionally, information on the baseline degree of cerebrovascular status measured by preimplantation brain imaging with focus on cerebral small vessel disease, brain atrophy, and silent brain infarctions together with serum neurochemical biomarkers and cognition test results before device implantation would enable inferences on chronic cerebral injuring processes due to CHF disease as opposed to (sub)acute injury potentially attributed to device therapy.

However, this demands at best brain magnetic resonance or at least computed tomography imaging before LVAD implantation, which, because it is not a mandatory presurgery investigation, would require a longitudinal study design with imaging acquisitions additional to clinical indications.

Although in SPM analyses, a *P* value of 0.001 uncorrected for multiple testing reduces the risk for false‐negative results compared with a *P* value of 0.05 with FWE correction, conversely it buries the risk for false positivity. This might be reasonable in small explorative studies but should be considered carefully with larger sample sizes.

Lastly, because the control subjects used for SPM analyses were selected retrospectively, biomarker levels of healthy controls are missing. Therefore, our data were interpreted in regard to healthy control groups or cohorts with HF from current literature.

## CONCLUSIONS

^18^F‐FDG‐PET and neurochemical serum markers are promising tools to investigate cerebral injury in patients with HF treated with LVAD or conservative therapy, who often present cognitive impairment and pathological biomarker levels. Indications for subtle cerebral hypometabolism were found in explorative SPM analyses using uncorrected thresholds. Given the longer wearing times of modern LVAD devices, our preliminary findings should encourage longitudinal studies to evaluate their impact on real‐world management in order to boost early prevention of cognitive decline.

## Sources of Funding

Maria M. Gabriel was funded by the Ellen‐Schmidt‐Scholarship of Hannover Medical School. Lorenzo Barba was funded by the Medical Faculty of Martin‐Luther‐University Halle‐Wittenberg (Junior Clinician Scientist Program No. JCS24/02).

## Disclosures

Rudolf A. Werner has received speaker honoraria from Novartis/AAA and PentixaPharm, reports advisory board work for Novartis/AAA and Bayer. The remaining authors have no disclosures to report.

## Supporting information

Supplemental Methods and Results Data S1–S3
Tables S1–S3
Figures S1–S2
References 77–82

STROBE Statement
