# Supplementary material for: Cerebral Injury and Impaired Brain Metabolism in Patients With Left Ventricular Assist Device and Chronic Heart Failure —An Interdisciplinary Cognition, Blood‐Based Biomarker and Brain Metabolism Assessment
Source: J Am Heart Assoc. 2026 Sep 9;15(19):e043727. doi: 10.1161/JAHA.125.043727 (PMC13745000; doi:10.1161/JAHA.125.043727)
Supplement: Supplementary file 1 [file JAH3-15-e043727-s001.pdf]

STROBE Statement—checklist of items that should be included in reports of observational studies

	Item No.	Recommendation	Page No.	Relevant text from manuscript
**Title and abstract**	1	(*a*) Indicate the study's design with a commonly used term in the title or the abstract	5	"In this cross-sectional pilot study […]"
		(*b*) Provide in the abstract an informative and balanced summary of what was done and what was found	5	We prospectively enrolled 19 heart failure patients with LVAD support and 13 advanced chronic heart failure (CHF) patients receiving best medical treatment […]. Of all patients, 69% showed cognitive dysfunction (…).
**Introduction**				
Background/rationale	2	Explain the scientific background and rationale for the investigation being reported	8	"There is evidence for stabilization or improvement of cognitive capabilities following LVAD implantation, especially in the first year [10–16]. However, some studies report postoperative cognitive decline in LVAD patients [13,17]. […] Yet, previous studies identified structural cerebral changes such as a high burden of cerebral microbleeds and brain atrophy [18–21] as well as a variety of factors altering brain physiology, attributed to LVAD therapy. These are so far of

				rather hypothetical character, but include endothelial dysfunction, permanently increased sympathetic activation, abnormal cerebral autoregulation, diastolic hypertension and a lack of cerebral clearance potentially leading to accumulation of β-amyloid [9,22,23]."
Objectives	3	State specific objectives, including any prespecified hypotheses	8f	" Yet, previous studies identified structural cerebral changes such as a high burden of cerebral microbleeds and brain atrophy [18–21] as well as a variety of factors altering brain physiology, attributed to LVAD therapy. […] In this exploratory study, we aimed to assess neurocognitive performance of patients under long-term LVAD therapy and patients with conservatively treated chronic heart failure (CHF) under best medical treatment and for the first time relate the results to 1) serum biomarkers of neuroaxonal, and astrocytic activation as well as neurodegenerative markers (i.e. neurofilament light chain protein (NfL), phosphorylated

				Tau protein at threonine 181 (pTau181) and glial fibrillary acidic protein) and 2) cerebral glucose metabolism, quantified by ^{18}F-FDG-PET/CT"
**Methods**				
Study design	4	Present key elements of study design early in the paper	10	"Between September 2023 and May 2024, 32 HF patients […] were prospectively enrolled into the study."
Setting	5	Describe the setting, locations, and relevant dates, including periods of recruitment, exposure, follow-up, and data collection	10f	Between September 2023 and May 2024, 32 HF patients treated at the Department of Heart-, Thoracic-, Transplantation and Vascular Surgery or the Department of Cardiology at Hannover Medical School were prospectively enrolled into this explorative cohort study. […] All patients underwent whole-body static ^{18}F-FDG-PET/CT […]"
Participants	6	(*a*) *Cohort study*—Give the eligibility criteria, and the sources and methods of selection of participants. Describe methods of follow-up<br>*Case-control study*—Give the eligibility criteria, and the sources and methods of case ascertainment and control selection. Give the rationale for the choice of cases and controls<br>*Cross-sectional study*—Give the eligibility criteria, and the sources and methods of selection of participants	9-11	Cross-sectional study: "Patients were included when having an age over 18 years and a left ventricular ejection fraction below 35%. We excluded all patients suffering from a malignant disease, a chronic disease of the central nervous

system (e.g. a history of MCI or dementia), or a current sepsis according to the in-house standard operating procedure [29] to eliminate competing causes for cognitive impairment respectively cerebro-metabolic alterations. Since the HeartWare HVAD System (HVAD, Medtronic) is known to be far less hemocompatible than the Heart Mate 3 (HM3, Abbott) [30], we only included patients with the latter device and excluded all patients with HVAD accordingly. […] A head imaging-confirmed history of stroke was no exclusion criterion as strokes are a potential complication of LVAD therapy [30–33], and therefore excluding these patients would present a selection bias." "All patients underwent whole-body static ^{18}F-FDG-PET/CT […]" For the brain metabolism analysis in FDG PET/CT, the patient results were also compared with a healthy control group: "We therefore retrospectively selected sixteen age-adjusted subjects without

				cardiac and neurological disease including 5 men and 11 women (median age 62 years (49-66)) who underwent ^{18}F-FDG-PET/CT with unremarkable findings using the same imaging protocol and the same scanner as the heart failure patients
		(*b*) *Cohort study*—For matched studies, give matching criteria and number of exposed and unexposed<br>*Case-control study*—For matched studies, give matching criteria and the number of controls per case		NA
Variables	7	Clearly define all outcomes, exposures, predictors, potential confounders, and effect modifiers. Give diagnostic criteria, if applicable	10-12	"A head imaging-confirmed history of stroke was no exclusion criterion as strokes are a potential complication of LVAD therapy [30–33], and therefore excluding these patients would present a selection bias. […] Clinical characteristics including the length of LVAD support, the underlying heart disease, cardiovascular risk factors, routine laboratory parameters, and concomitant complications such as current infections were recorded. Neurological status and functional capacity were evaluated using the National Institutes of Health Stroke Scale (NIHSS) and modified Rankin

scale (mRS). Montreal Cognitive Assessment (MoCA) [34,35] was used to evaluate cognitive function. […] we additionally assessed the Frontal Assessment Battery (FAB) [37] in all patients to evaluate frontal lobe dysfunction. […] The study participants were screened for depression. […]Opening characteristics of the aortic valve (AV) during contraction was retrospectively acquired from the latest echocardiography before study inclusion […] Image analysis was performed using 1) single subject vs. control group (SSVCG) analyses to detect metabolic alterations i.e. hypo- and hypermetabolic clusters of cohesive voxels in each patient individually, and 2) SPM's two-sample t test for group comparisons to determine differences between the different patient groups (a P-value <0.001 uncorrected for multiple testing and no voxel threshold was applied for both contrasts, hypo- and hypermetabolism). […] Serum

				biomarkers were quantified with commercially purchased immunoassays”
Data sources/ measurement	8*	For each variable of interest, give sources of data and details of methods of assessment (measurement). Describe comparability of assessment methods if there is more than one group	10-13	“Clinical characteristics including the length of LVAD support, the underlying heart disease, cardiovascular risk factors, routine laboratory parameters, and concomitant complications such as current infections were recorded. **Neurological status** and functional capacity were evaluated using the National Institutes of Health Stroke Scale (NIHSS) and modified Rankin scale (mRS). […] the Montreal Cognitive Assessment (MoCA) [34,35] was used to evaluate cognitive function. […], we additionally assessed the Frontal Assessment Battery (FAB) [37] in all patients to evaluate frontal lobe dysfunction.” **“Opening characteristics of the aortic valve** (AV) during contraction was retrospectively acquired from the latest echocardiography before study inclusion” **FDG-PET/CT**: “A healthy

				control group is mandatory in the SPM analyses. We therefore retrospectively selected sixteen age-adjusted subjects without cardiac and neurological disease including 5 men and 11 women (median age 62 years (49-66)) who underwent ^{18}F-FDG-PET/CT with unremarkable findings using the same imaging protocol and the same scanner as the heart failure patients […] The detailed procedure is enclosed in the supplements as well as further information on the workflow of ^{18}F-FDG-PET/CT acquisition, data pre-processing, and image analysis in order to obtain voxel values for all hypometabolic clusters. […] **Serum biomarkers** were quantified with commercially purchased immunoassays […]"
Bias	9	Describe any efforts to address potential sources of bias	9-12, 14, 19	"We excluded all patients suffering from a malignant disease, a chronic disease of the central nervous system (e.g. a history of MCI or dementia), or a current sepsis according to the in-house standard operating procedure [29] to exclude competing causes for cognitive

impairment respectively cerebro-metabolic alterations. […] Clinical characteristics including the length of LVAD support, the underlying heart disease, cardiovascular risk factors, routine laboratory parameters, and concomitant complications such as current infections were recorded. […]We therefore retrospectively selected sixteen age-adjusted subjects without cardiac and neurological disease including 5 men and 11 women (median age 62 years (49-66)) who underwent ^{18}F-FDG-PET/CT with unremarkable findings using the same imaging protocol and the same scanner as the heart failure patients. […] A P-value <0.001 uncorrected for multiple testing was applied […] Linear regression models were conducted to adjust the outcome variables - in deference of the small sample size of n=32 limited to 3 variables - for the most significant confounders (i.e. age, arterial hypertension, history of stroke). The Benjamini-Hochberg method

was applied to adjust the P value in order to reduce the bias caused by multiple tests. […] Prescribed medication potentially affecting glucose metabolism according to the EANM procedure guidelines for brain PET imaging are attached in the supplements [24].”; “At first, when interpreting the PET-findings, it should be considered that utilising an uncorrected P-value without multiplicity correction carries the high risk of false positive results. Therefore, positive, i.e. hypometabolic, findings should not be overstated but rather considered alongside the other outcome variables.

A P-value <0.05 with family-wise error (FWE) correction presents a more rigorous threshold ideally reducing the risk for false-positives, potentially explaining the extensive reduction of detected significant hypometabolism in our results. However false negative results after FWE correction cannot be excluded either, indicated for example by

				the missing detection of an MRI-validated infarct core (see Supplemental Materials, Figure S1 and S2, Table S2). Further verification was not feasible missing dynamic data. Thus, and in regard of the study's exploratory approach, we decided on applying the threshold with lesser evidence for false results."
Study size	10	Explain how the study size was arrived at	10, 13	"Due to the exploratory nature of the study and the lack of comparable data in the literature, a formal sample size calculation was not feasible; the university's institute for biometrics was involved during protocol development. […] We screened 40 advanced HF patients in total who were scheduled for ^{18}F-FDG-PET/CT, of whom 32 were recruited (…)"

Continued on next page

Quantitative variables	11	Explain how quantitative variables were handled in the analyses. If applicable, describe which groupings were chosen and why	12	“Categorical variables are reported in frequencies and continuous variables as median with interquartile range (IQR)”
Statistical methods	12	(*a*) Describe all statistical methods, including those used to control for confounding	12f	“Statistical analyses were performed using IBM SPSS-Statistics for Windows (v.29.0.1.0; IBM-Deutschland) and R studio (v.4.5.1; R Foundation, Vienna, Austria). R studio and SPM were also utilized to visually present data when necessary. Graphical abstract was created with BioRender.com. Categorical variables are reported in frequencies and continuous variables as median with interquartile range (IQR). In univariate analysis of baseline characteristics, the Pearson Chi-square test was applied for categorical variables. Group differences in continuous variables were evaluated using Mann–Whitney test for independent samples. Confidence intervals (CI) were calculated using Hodges-Lehmann estimator. Correlation analysis was performed with the Spearman’s rank correlation coefficient to identify similarities between neurodegenerative

			outcome parameters (cerebral hypometabolic volume, neurocognitive test results, biomarker assessments) and clinical measurements. The Kruskal-Wallis test was used to compare the categories of AV opening on the outcome parameters. Linear regression models were conducted to adjust the outcome variables - in deference of the small sample size of n=32 limited to 3 variables - for the most significant confounders (i.e. age, arterial hypertension, history of stroke).<br>The Benjamini-Hochberg method was applied to adjust the P value in order to reduce the bias caused by multiple tests. The regression models are likely overfitted and lack statistical robustness due to the low ratio of event to variables, and their results should therefore be considered with caution. Statistical significance was defined as a P value of <0.05.”
	(*b*) Describe any methods used to examine subgroups and interactions	12f	“Group differences in continuous variables were evaluated using Mann–Whitney test for independent samples. […] Correlation analysis was performed with the Spearman’s rank

			correlation coefficient to identify similarities between neurodegenerative outcome parameters (cerebral hypometabolic volume, neurocognitive test results, biomarker assessments) and clinical measurements. The Kruskal-Wallis test was used to compare the categories of AV opening on the outcome parameters. Linear regression models were conducted to adjust the outcome variables - in deference of the small sample size of n=32 limited to 3 variables - for the most significant confounders (i.e. age, arterial hypertension, history of stroke)."
	(*c*) Explain how missing data were addressed	10, 16	"One CHF patient only received myocardial ^{18}F-FDG-PET/CT instead of a whole-body scan and therefore could not be included in the ^{18}F-FDG-PET/CT analyses of the brain, but for further analyses […]Serum samples were available from all 32 HF patients, with just one missing value of a LVAD patient in the GFAP immunoassay."
	(*d*) *Cohort study*—If applicable, explain how loss to follow-up was addressed<br>*Case-control study*—If applicable, explain how matching of cases and controls was addressed<br>*Cross-sectional study*—If applicable, describe analytical methods taking account of sampling strategy	NA	

	(*e*) Describe any sensitivity analyses	19	"At first, when interpreting the PET-findings, it should be considered that utilising an uncorrected P-value without multiplicity correction carries the high risk of false positive results. Therefore, positive, i.e. hypometabolic, findings should not be overstated but rather considered alongside the other outcome variables.<br>A P-value <0.05 with family-wise error (FWE) correction presents a more rigorous threshold ideally reducing the risk for false-positives, potentially explaining the extensive reduction of detected significant hypometabolism in our results. However false negative results after FWE correction cannot be excluded either, indicated for example by the missing detection of an MRI-validated infarct core (see Supplemental Materials, Figure S1 and S2, Table S2). Further verification was not feasible missing dynamic data. Thus, and in regard of the study's exploratory approach, we decided on applying the threshold with lesser evidence for false results."

**Results**				
Participants	13*	(a) Report numbers of individuals at each stage of study—eg numbers potentially eligible, examined for eligibility, confirmed eligible, included in the study, completing follow-up, and analysed	10, 13	"One CHF patient only received myocardial ^{18}F-FDG-PET/CT instead of a whole-body scan and therefore could not be included in the ^{18}F-FDG-PET/CT analyses of the brain, but for further analyses […] We screened 40 advanced HF patients in total who were scheduled for ^{18}F-FDG-PET/CT, of whom 32 were recruited (two patients suffered from a sepsis at time of ^{18}F-FDG-PET/CT, 3 were implanted with a HVAD and three declined study inclusion)."
		(b) Give reasons for non-participation at each stage	10, 13, 16	"One CHF patient only received myocardial ^{18}F-FDG-PET/CT instead of a whole-body scan and therefore could not be included in the ^{18}F-FDG-PET/CT analyses of the brain, but for further analyses. […] We screened 40 advanced HF patients in total who were scheduled for ^{18}F-FDG-PET/CT, of whom 32 were recruited (two patients suffered from a sepsis at time of ^{18}F-FDG-PET/CT, 3 were implanted with a HVAD and three declined study inclusion). […] Serum samples were available from all 32 HF patients, with just one

				missing value of a LVAD patient in the GFAP immunoassay. ”
		(c) Consider use of a flow diagram	Graphical abstract	
Descriptive data	14*	(a) Give characteristics of study participants (eg demographic, clinical, social) and information on exposures and potential confounders	13	“Demographics, clinical characteristics and neurocognitive/biomarker assessment results are presented in Table 1. Prescribed medication potentially affecting glucose metabolism according to the EANM procedure guidelines for brain PET imaging are attached in the supplements [24]. [… LVAD patients were in median 59 (IQR: 52-66) and CHF patients 47 (40-64) years old. Sixteen (84.2%) LVAD and 9 (69.2%) CHF patients were male, accordingly 3 (15.8%) LVAD and 4 (30.8%) CHF patients were female.”
		(b) Indicate number of participants with missing data for each variable of interest	10, 14, 16	“One CHF patient only received myocardial ^{18}F-FDG-PET/CT instead of a whole-body scan and therefore could not be included in the ^{18}F-FDG-PET/CT analyses of the brain, but for further analyses. […]The AV of another six patients opened never or just seldom, and four patients could not be categorized. […] Serum samples

				were available from all 32 HF patients, with just one missing value of a LVAD patient in the GFAP immunoassay"
		(c) *Cohort study*—Summarise follow-up time (eg, average and total amount)	NA	
Outcome data	15*	*Cohort study*—Report numbers of outcome events or summary measures over time	NA	
		Case-control study—Report numbers in each exposure category, or summary measures of exposure	NA	
		Cross-sectional study—Report numbers of outcome events or summary measures	14f	"Median MoCA scores were 25 points (IQR: 23-26) in the LVAD group and 25 points (IQR: 21,5-26,5) in the CHF group, with 68.4% (LVAD group) and 69.5% (CHF group) of the patients having a MoCA score <26 (i.e. MCI). […]The SPM group comparison of LVAD and CHF patients against the 16 control subjects revealed a frontotemporally accentuated cerebral hypometabolism in both - LVAD and CHF patients (p<0.001 uncorr.) (Figure 4A and 4C). The frontotemporal hypometabolism was less pronounced in CHF patients (4C) than in the LVAD patients (4A)."
Main results	16	(*a*) Give unadjusted estimates and, if applicable, confounder-adjusted estimates and their precision (eg, 95% confidence interval). Make clear which confounders were adjusted for and why they were included	14-17	**Neurocognitive functions** Median MoCA scores were 25 points (IQR: 23-26) in the LVAD group and 25 points (IQR: 21,5-26,5) in the CHF group, with 68.4% (LVAD group) and 69.5%

(CHF group) of the patients having a MoCA score <26 (i.e. MCI). The MCI severity category ranges were spread similarly in both groups (p=0.91), which is reflected by the group comparison (p=0.842 [95% CI, -2-2]) (Figure 2A). The results of patients with a history of stroke did not differ to those without (p = 0.714 [95% CI, -3-2]) (Figure 2B). The FAB results revealed frontotemporal impairment in five patients: n=3 with LVAD and n=2 with CHF. The two groups did not show significant differences in the FAB results (p= 0.368 [95% CI, -1-2]) (Figure 2C), even after excluding all patients with a history of stroke (p=0.637 [95% CI, -1-2]) (Figure 2D) (3 of the 10 patients with a history of stroke had a residing infarct core in the frontal lobe). […]

Cerebral glucose metabolism

[…] The LVAD group revealed cerebral metabolic alterations with a median total hypometabolic volume (THV) of 1.03 ml (IQR: 0-7.34 ml), which was significantly greater than in the CHF group (0 ml, IQR: 0-0.1 ml; p=0.011 [95% CI, -6.128-0]) (Figure 5A). The

difference barely misses significance after excluding all patients with a history of stroke (LVAD: 0.69 ml (0-5.28), CHF: 0 ml (0-0); p=0.051[95% CI, -1.576-0]), though stroke lesions produce partially large hypometabolic clusters (Figure 5B).

No significant effect of age on THV could be seen in the whole LVAD (Spearman's ρ=0.141 [95% CI -0.347-0.569], p=0.564) nor CHF group (ρ=0.138 [95% CI -0.488- 0.67], p=0.669). However, excluding all patients with a history of stroke revealed a significant association between age and THV in LVAD patients (ρ=0.634 [95% CI -0.015- 0.907], p=0.049).

The correlation analyses lack statistical power due to the small sample size. The significant difference in THV is more likely to be attributed to the substantial differences in age and cardiovascular risk factors than device therapy."[…]

Linear regression models were tested for each outcome variable adjusted for the most significant confounders i.e. age, arterial hypertension and history of stroke.

			The only significant result was an independent effect of history of stroke on the THV in the whole HF cohort (corrected $R^2=0.274$, $\beta=14.86$, [95% CI 5.796-23.93], $p=0.002$). All other regression models missed the significance threshold after correction for multiple testing.”
	(*b*) Report category boundaries when continuous variables were categorized	14	“Out of the nineteen HM3 patients six (31.6%) patients had an AV opening at (almost) every contraction and three (15.8%) patients had their AV opening occasionally during the ejection phase. The AV of another six (31.6%) patients opened never or just seldom, and four (21.1%) patients could not be categorized.”
	(*c*) If relevant, consider translating estimates of relative risk into absolute risk for a meaningful time period	NA	

Continued on next page

Other analyses	17	Report other analyses done—eg analyses of subgroups and interactions, and sensitivity analyses	14, 16, 17, 19	"Kruskal-Wallis test did not show a significant difference for any of our neurological outcome parameters. […] Associations of impaired brain metabolism, serum biomarkers of neuronal injury/activation and cognitive performance are attached in the supplements. Linear regression models were tested for each outcome variable adjusted for the most significant confounders i.e. age, arterial hypertension and history of stroke. The only significant result was an independent effect of history of stroke on the THV in the whole HF cohort (corrected R^2=0.274, β=14.86, [95% CI 5.796-23.93], p=0.002). All other regression models missed the significance threshold after correction for multiple testing. […]At first, when interpreting the PET-findings, it should be considered that utilising an uncorrected P-value without multiplicity correction carries the high risk of false positive results. Therefore, positive, i.e. hypometabolic, findings should not be overstated but rather considered alongside the other outcome

				variables.<br>A P-value <0.05 with family-wise error (FWE) correction presents a more rigorous threshold ideally reducing the risk for false-positives, potentially explaining the extensive reduction of detected significant hypometabolism in our results. However false negative results after FWE correction cannot be excluded either, indicated for example by the missing detection of an MRI-validated infarct core (see Supplemental Materials, Figure S1 and S2, Table S2). Further verification was not feasible missing dynamic data. Thus, and in regard of the study's exploratory approach, we decided on applying the threshold with lesser evidence for false results."
**Discussion**				
Key results	18	Summarise key results with reference to study objectives	17f	"We conducted this prospective exploratory study of 32 severe HF patients – 19 with LVAD and 13 without - to further investigate the cerebral impairment in HF patients after LVAD implantation by 1) cognitive testing, 2) cerebral glucose metabolism measured by 18F-FDG-PET/CT and 3) serum

				biomarkers of neuronal and astrocytic injury.<br>Few previous studies investigated cerebral metabolic changes using 18F-FDG-PET [6,37,50,51] or neuronal/astrocytic serum biomarkers [44–47] in HF patients. To our knowledge, studies on neurocognitive outcome in LVAD patients have been limited to cognitive testing, thus we present the first study, which investigated cerebral metabolism and serum neuronal and astroglial markers in LVAD patients.<br>The main findings of our investigation were i) diminished cognitive capabilities in severe HF patients, ii) elevated serum biomarker levels and iii) indications of a frontotemporally accentuated cerebral hypometabolism independent of a history of stroke in both groups of severe HF patients - namely in those with and those without mechanical circulatory support."
Limitations	19	Discuss limitations of the study, taking into account sources of potential bias or imprecision. Discuss both direction and magnitude of any potential bias	18-29, 23-24	"However, an association between FDG metabolism and cognitive performance should be regarded with caution for several reasons: (…) […]At first, when interpreting

the PET-findings, it should be considered that utilising an uncorrected P-value without multiplicity correction carries the high risk of false positive results. Therefore, positive, i.e. hypometabolic, findings should not be overstated but rather considered alongside the other outcome variables.

A P-value <0.05 with family-wise error (FWE) correction presents a more rigorous threshold ideally reducing the risk for false-positives, potentially explaining the extensive reduction of detected significant hypometabolism in our results. However false negative results after FWE correction cannot be excluded either, indicated for example by the missing detection of an MRI-validated infarct core (see Supplemental Materials, Figure S1 and S2, Table S2). Further verification was not feasible missing dynamic data. Thus, and in regard of the study's exploratory approach, we decided on applying the threshold with lesser evidence for false results. Due to the shortage of donor organs, heart transplantation is preferably offered

patients with good physical constitution facilitating survival of the transplantation surgery and following immunosuppression. This is often accompanied by a younger chronological age and a lower cardiovascular burden. . However, these HF patients were the only ones regularly receiving whole-body FDG-PET imaging.

The greater baseline vascular burden in the LVAD patients not only contributes to higher prevalence of cerebral strokes. It leads in smaller scale to cerebral small vessel disease as well as microvascular hypoperfusion and consequently neuronal damage [5,7–9] and hypometabolism [51]. Therefore, the greater THV measured in the LVAD group might be partially based on the worse state of cerebral perfusion following the greater vascular burden.

Moreover, it should be discussed whether more severely affected CHF patients with LVEF below 25% could serve as a more compatible control group when tolerating the 18F-FDG-PET/CT examination, especially since an effect of the LVEF on brain

metabolism has been demonstrated in the past [37,59].

Our data present a considerable discrepancy between the SSVCG analyses that detect – apart from larger infarct cores - discreet hypometabolic defects opposing to the more widespread reduction in brain metabolism, particularly frontotemporally, seen in the group comparisons. We hypothesize that the metabolic derogation affecting most of the cerebral grey matter areas due to cerebral microangiopathy and thus hypoperfusion is too subtle to be detected in SSVCG analyses, which is why SPM group comparisons are needed to raise the frontotemporal hypometabolism to detectability. The apparent difference there between the LVAD and CHF group might be at least partially attributed to the greater vascular burden in our LVAD patients, as discussed above.

(…)

The influence of duration of device support, the device type itself and the status of remaining AV opening capabilities still remains poorly investigated. […] Hence, the interpretation of pTau181 levels in

CHF patients may be not univocal.
[…] First, the small sample size limits the generalizability of our results. For the same reason, analyses on patient subgroups with testing for e.g. the extent of driveline infection, AV opening characteristics or aetiological cardiovascular pathology were greatly hampered. Investigating sex or gender specific differences was likewise limited to descriptive measurements.
The considerable difference in age and cardiovascular risk factors limits the comparability of our two groups as an age difference of about 12 years and a higher vascular burden between LVAD and CHF groups are potential confounders for brain metabolism, cognitive performance and biomarkers of cerebral injury. Due to the small sample size, sufficient multivariate models adjusting for potential confounders were limited to three independent variables. Additionally, since the healthy control groups' age distribution matches the LVAD group reasonably well, the risk for false negative findings in the CHF group is to be considered. Hence,

our findings should be interpreted as exploratory, hypothesis-generating, and requiring extensive validation.

The cross-sectional design lacks comparable pre-LVAD implantation as well as longitudinal follow-up data, and thus misses information on dynamics of the measured parameters. Additionally, information on the baseline degree of cerebrovascular status measured by pre-implantation brain imaging with focus on cerebral small vessel disease, brain atrophy and silent brain infarctions together with serum neurochemical biomarkers and cognition test results before device implantation would enable inferences on chronic cerebral injuring processes due to chronic heart failure disease oppose to (sub)acute injury potentially attributed to device therapy. However, this demands at best brain magnetic resonance or at least computed tomography imaging before LVAD implantation which, since it is not a mandatory pre-surgery investigation, would require a longitudinal study design with imaging acquisitions additional to

				clinical indications.<br>Although in SPM analyses, a P value of 0.001 uncorrected for multiple testing reduces the risk for false-negative results compared to a P value of 0.05 with FWE correction, conversely it buries the risk for false positivity. This might be reasonable in small explorative studies but should be considered carefully with larger sample sizes.<br>Lastly, since the control subjects used for SPM analyses were selected retrospectively, biomarker levels of healthy controls are missing. Therefore, our data was interpreted in regard to healthy control groups or heart failure cohorts from current literature.”
Interpretation	20	Give a cautious overall interpretation of results considering objectives, limitations, multiplicity of analyses, results from similar studies, and other relevant evidence	18-22	“The most frequently impaired cognitive functions in our patient groups - short- and long-term memory retrieval, verbal fluency and attention (Figure 3) – are related to the affected cerebral regions seen in the group comparisons i.e. merely frontal and temporal lobe (Figure 4). These findings correspond to recent studies that found cerebral metabolic impairments in concordant frontal [51,52] and

temporal [6,50–53] cognition-related regions in HF patients. These regions are assumed to be primarily affected by hypometabolism due to a high susceptibility to hypoperfusion and hypoxia [50,52,53] or as a possible correlate of concomitant depressive symptoms [51].

However, an association between FDG metabolism and cognitive performance should be regarded with caution for several reasons: (…) […] Most of our patients experienced their stroke more than one year prior to study inclusion. It is hence possible that elevated NfL concentrations may reflect chronic neuronal damage in its multifactorial nature being associated for example with chronic cerebral vascular burden due to continuous microbleeds and silent strokes [66–68], impaired cerebral perfusion, proinflammatory disposition, endothelial dysfunction and neurohumoral dysregulation [8,46,69,70]. […] [GFAP's] increased expression can indicate reactive astrogliosis due to neighboring neuronal death as well as blood-brain barrier disruption [71]. For HF

				patients, factors such as cerebral hypoperfusion, hypoxia, and neuroinflammation may contribute to this elevation. […] On the one hand, the longitudinal increase of pTau181 levels over years may suggest beginning CHF-related neurodegenerative processes [45], also in the context of AD-like patterns. On the other hand, given the influence of comorbidities on pTau181 levels such as renal disease, it is possible that pTau181 levels in CHF patients may not be strictly associated with incipient AD.”
Generalisability	21	Discuss the generalisability (external validity) of the study results	20,23	“The influence of duration of device support, the device type itself (..) still remains poorly investigated. Of the two dominantly implanted devices in recent years, the centrifugal flow HVAD system presented a higher risk of strokes compared with the HM3 device [34], consequently leading to its end of distribution in 2021. Additionally, the HVAD is known to be less hemocompatible with higher levels of hemolysis compared to the HM3 [30]. Therefore, HVAD patients may have an increased susceptibility for

				subclinical brain injury e.g. cerebral microbleeds - conceivably comparable to hemolytic anemia such as sickle-cell disease [60]- that could cause over time manifest cognitive symptoms [13]. The other centrifugal-flow pump design HM3 aims for better hemocompatibility through magnetic levitation of the pump, wider blood-flow pathways as well as an artificial pulse unsynchronized to the heart rate [61] and thus has a different effect on the vascular physiology. […] First, the small sample size limits the generalizability of our results. For the same reason, analyses on patient subgroups with testing for e.g. extent of driveline infection, AV opening characteristics or etiological cardiovascular pathology were greatly hampered. Investigating sex or gender specific differences was likewise limited to descriptive measurements.”
**Other information**				
Funding	22	Give the source of funding and the role of the funders for the present study and, if applicable, for the original study on which the present article is based	24	“MMG was funded by the Ellen-Schmidt-Scholarship of Hannover Medical School. LB was funded by the Medical Faculty of Martin-

	Luther-University Halle-Wittenberg (Junior Clinician Scientist Program No. JCS24/02)."

*Give information separately for cases and controls in case-control studies and, if applicable, for exposed and unexposed groups in cohort and cross-sectional studies.

Note: An Explanation and Elaboration article discusses each checklist item and gives methodological background and published examples of transparent reporting. The STROBE checklist is best used in conjunction with this article (freely available on the Web sites of PLoS Medicine at http://www.plosmedicine.org/, Annals of Internal Medicine at http://www.annals.org/, and Epidemiology at http://www.epidem.com/). Information on the STROBE Initiative is available at www.strobe-statement.org.
