# Supplementary material for: Cerebral Injury and Impaired Brain Metabolism in Patients With Left Ventricular Assist Device and Chronic Heart Failure —An Interdisciplinary Cognition, Blood‐Based Biomarker and Brain Metabolism Assessment
Source: J Am Heart Assoc. 2026 Sep 9;15(19):e043727. doi: 10.1161/JAHA.125.043727 (PMC13745000; doi:10.1161/JAHA.125.043727)
Supplement: Supplementary file 2 [file JAH3-15-e043727-s002.pdf]

Supplemental Material

Supplemental Methods and Results

Data S1

Materials and methods

In case of LVAD infection based on ^{18}F-FDG-PET/CT results, the extent was evaluated by subdivision of the device into the four components (i.e. driveline entry point, subcutaneous driveline pathway, pump and outflow graft) according to the consensus-based standardization of definitions of infections in LVAD patients of the International Society of Heart and Lung Transplantation (ISHLT) [77].

Data S2

MoCA

The MoCA is a 30-point test with a cut-off score of 26 points indicating MCI [35] while less than 17 points indicate moderate to severe cognitive decline. MCI score ranges were defined according to Nasreddine et al. [35]. One point is added on the MoCA score if a patient has less than 12 years of education to account for the effect of poor education on cognitive capabilities [35]. One of our patients had severely limited visual function, thus the MoCA Blind version was performed, where the visuospatial and naming task [78] are omitted which results in a total score of 22 points which can be converted back to 30 by multiplying the obtained score with 30 and dividing the result by 22 (decimal digits are rounded).

FAB

The FAB is an 18-point test specifically designed for the assessment of frontal lobe functions [38]. We performed the German version FAB-D [79] and evaluated the patients test results using cut-off scores adjusted for age (grouped in 10 years-age classes between 50 and 95 years) and education (less than or equal and above 12 years). As proposed for this test, scores below the 5th percentile were considered abnormal indicating a patient being ‘slightly to severely

impaired'. Since nine of our patients were younger than 50 years, they were classified according to the 50-59 years age group.

Data S3

^{18}F-FDG-PET/CT – Image and SPM analysis

Patients were obliged to fast for at least 6 h before the examination. After confirmation that the patients' blood glucose level is below 8 mmol/L, a dose of 256 ± 57 MBq ^{18}F-FDG (3 MBq/kg body weight) was injected intravenously. The PET/CT acquisition was started 60 min after the injection, starting with a low-dose whole body CT for attenuation correction followed by the static PET scan. All CT studies were reconstructed using the iterative Ultra HD algorithm in combination with time-of-flight and point-spread function information (Siemens Healthineers; 2 iterations, 21 subsets, matrix 200, zoom 1.0; Gaussian filter 2.00). Within the control group known cardiovascular diseases, local or systemic cerebral atrophy in computed tomography of the brain and diagnosis of depression with need for antidepressants additionally served as exclusion criteria. As the control group subjects received not static but dynamic whole body ^{18}F-FDG-PET/CT, the 60 to 70 min frame out of the 90 min dynamic imaging ^{18}F-FDG-PET/CT acquisition was used in compliance with the static ^{18}F-FDG-PET/CT imaging where the acquisition in the craniocaudal direction is performed typically 60 min after tracer injection.

The images of all subjects, i.e. study patients and control group, were all reconstructed using the same iterative Ultra HD algorithm in combination with time-of-flight and point-spread function information as described above. However, the control group studies have been smoothed using a Gaussian filter of 5 mm full width at half maximum (FWHM). Consequently, the Gaussian smoothing factor of the images of our heart failure patients needed to be adjusted to match the applied smoothing of the control group images. According to Yu-Jin Zhang 'Two Gaussian functions can be cascaded, i.e. applied consecutively, to give

a Gaussian convolution result which is equivalent to a kernel with the variance equal to the sum of the variances of the constituting Gaussian kernels' [80].

This translates to our study as follows: The images of 31 heart failure patients which were smoothed with a Gaussian kernel of 2 mm were resmoothed with a kernel of 4.5 mm, since the variances of 2 (i.e. 4) and 4.5 (i.e. 20.25) sum up to approximately 25, the variance of 5 which is the FWHM of the Gaussian kernel applied for smoothing to the scans of the healthy control group.

In preparation for PET image analysis, the brain is cropped out of the whole-body PET/CT DICOM images and afterwards resmoothed with a Gaussian kernel of 4.5 mm using PMOD Version 2.55 (PMOD Technologies Ltd., Zurich, Switzerland). SPM8 implemented in Matlab Version 8.4.0.150421 (R2014b) (The Mathworks Inc., Natick, MA, USA) was then applied for spatial normalization using a nonlinear registration algorithm by means of SPM, intensity normalization based on the pons as a reference region as well as the image analysis.

Subsequently to the SPM analysis, we applied the automated-anatomical labelling (AAL)-atlas, published by Tzourio-Mazoyer et al. [81] using PMOD Version 4.3 (PMOD Technologies Ltd., Zurich, Switzerland). This allowed us to obtain the total number of significant voxels as well as regional mapping of the clusters in accordance with the atlas's volumes of interest.

In advance of applying the AAL-atlas, separately for both contrasts (i.e. hypo- and hypermetabolism) all voxel values below a certain T-statistic (i.e. the corresponding T-statistic to a P-value <0.001, depending on the conducted statistical analysis (e.g. SSVCG, two-sample t test)) needed to be replaced by 0 and voxel values greater or equal the T-statistic were replaced with 1 (based on peak-level significance).

A T-statistic of 3.732834 was used for the SSVCG analyses. Since a voxel in SPM has a defined volume of 8 mm^3, dividing the total number of e.g. hypometabolic voxels of a brain with 125, converts the number of voxels into a volume in ml.

P-value for SPM analyses

A P-value <0.001 uncorrected for multiple comparisons is commonly used in exploratory imaging studies but carries the risk for false-positive results due to the lack of correction for multiple testing. The alternative widely used method is a P-value <0.05 with family-wise error correction (FWE).

We decided to use the P-value <0.001 uncorr. since the FWE correction potentially leads to extensive false-negative results.

As depicted in Figure S1, the FWE correction leads to an excessive reduction in significant hypometabolic clusters in the group comparisons.

Similar results can be seen in the SSVCG analysis of one of our LVAD patients with a history of stroke. Strokes lead to diminished brain metabolism in irreversibly injured brain tissue (i.e. the infarct core) [82]. Most of the infarct core (i.e. the black wedge in the left frontal lobe in the PET images) is missing significant hypometabolism in the p <0.05 FWE corrected images as compared to the uncorrected p<0.001 images (Figure S2). For a detailed comparison of the different P values of all relevant hypometabolic clusters of our patients, see Table S1.

b) In the light of the cognitive impairment measured in the study patients, the extent of hypometabolism in the group comparisons after FWE correction appears biased by false-negative results as well. Cognitive impairment in the study patients measured by screening tests does not correspond with the almost unaffected brain metabolism according to FWE corrected group comparisons.

Results

Spearman Correlations of brain metabolism, neurochemical biomarkers, cognitive test results and clinical parameters

A significant association after Bonferroni correction could only be reported in LVAD patients between age and NfL levels (ρ=0.673 [95% CI 0.301-, 0.867] $p<0.001$). Although no significant age difference in LVAD patients with (median 58 (IQR: 51.5-65.5)) and without a history of stroke (median (IQR: 59 (52-66)) existed (p=0.968 [95% CI, -9-8]. No other significant correlation could be reported between any of the tested variables.

Table S1: Cluster and peak level p values of SPM SSVCG analyses listed for each patient's individual hypometabolic cluster(s).

		Cluster level			*Peak level*	
ID	*Cohort*	*kE*	*p FWE corr (corrected)*	*p uncorr*	*p FWE corr*	*p uncorr*
Patients with a history of stroke						
02	LVAD	1028	0.036	0.02	0.021	<0.001
03	LVAD	4836	<0.001	<0.001	0.001	<0.001
05	LVAD	136	0.489	0.361	0.466	<0.001
06	LVAD	421	0.193	0.115	0.022	<0.001
10	CHF	4998	<0.001	<0.001	<0.001	<0.001
		2286	0.002	0.001	0.004	<0.001
12	LVAD	3216	<0.001	<0.001	0.017	<0.001
16	LVAD	4313	<0.001	<0.001	0.002	<0.001
		146	0.472	0.344	0.382	<0.001
32	LVAD	759	0.073	0.041	0.036	<0.001
Patients without a history of stroke						
13	LVAD	178	0.423	0.296	0.207	<0.001
14	LVAD	2150	0.003	0.002	0.094	<0.001
		174	0.429	0.301	0.532	<0.001
		159	0.451	0.323	0.608	<0.001
23	LVAD	222	0.364	0.244	0.089	<0.001

Only patients were included with a minimum cluster size of 125 voxel (≙ 1ml). General level of significance was P < 0.001. Abbreviations: FWE family-wise error, kE number of voxels,

LVAD left ventricular assist device, CHF chronic heart failure, SPM Statistical parametric mapping, SSVCG single subject vs. control group.

Table S2: Serum biomarker concentrations of LVAD and CHF cohort of this study as well as comparative data from CHF and healthy controls according to current literature.

	LVAD Jungclaus et al.	*n*	*CHF Jungclaus et al.*	*n*	*CHF Traub et al.*	*n*	*Healthy controls*[*]	*n*
	median (IQR)		*median (IQR)*		*median (IQR)*		*median (IQR)/ mean ± SD*	
NfL (pg/ml)	44.5 (32-79)	19	48.4 (17.2-96)	13	26.4 (16.7-42)	146	19 (10-27)	1033
pTau181 (pg/ml)	22.1 (19.5-34.5)	19	25.7 (17.2-67.8)	13	1.57 (1.09-2.41)	146	9.4 ± 6	337
GFAP (pg/ml)	194.8 (126-299)	18	190.1 (130-231.2)	13	146 (165-384)	146	51.8 (41.2-69.7)	485

[*] The reference values of the healthy controls for the three serum biomarkers derive from cohorts originally published by Halbgebauer et al. [43] for NfL, Karikari et al. [48] for pTau181 and Meier et al. [40] for GFAP. Each biomarker has been quantified using the same analytical device as in this study – HD-X platform (Quanterix Inc., Lexington, USA) and Ella microfluidic system (Biotechne, Minneapolis, USA).

Data are presented as mean ± SD or median (IQR) as reported in the reference study.

Abbreviations: LVAD left ventricular assist device, CHF chronic heart failure, NfL neurofilament light chain protein, pTau181 phosphorylated Tau protein at threonine 181, GFAP glial fibrillary acidic protein, IQR interquartile range, SD standard deviation, BMI body mass index

Table S3: Prescribed medication in both cohorts potentially influencing regional metabolic rate of glucose consumption according to the EANM procedure guidelines for brain PET imaging using [^{18}F]-FDG, version 3 [24].

parameters	*LVAD*	*n*	*CHF*	*n*
Sedatives (Opioides)	2 (9.1%)	19	1 (7.7%)	13
Antidepressant Medication	4 (21.1%)	19	2 (15.4%)	13
Anticonvulsive Medication	3 (13.6%)	19	2 (15.4%)	13
Corticosteroids	0 (0%)	19	1 (7.7%)	13

Abbreviations: LVAD left ventricular assist device, CHF chronic heart failure, EANM European Association of Nuclear Medicine, PET positron emission tomography, [^{18}F]-FDG 18F-fluordeoxyglucose

Figure S1: Combined results of group comparisons of all 19 LVAD patients against the 16 control subjects for suprathreshold hypo- and hypermetabolism of SPM two-sample t-test in ^{18}F-FDG-PET/CT visualized as sectional images.

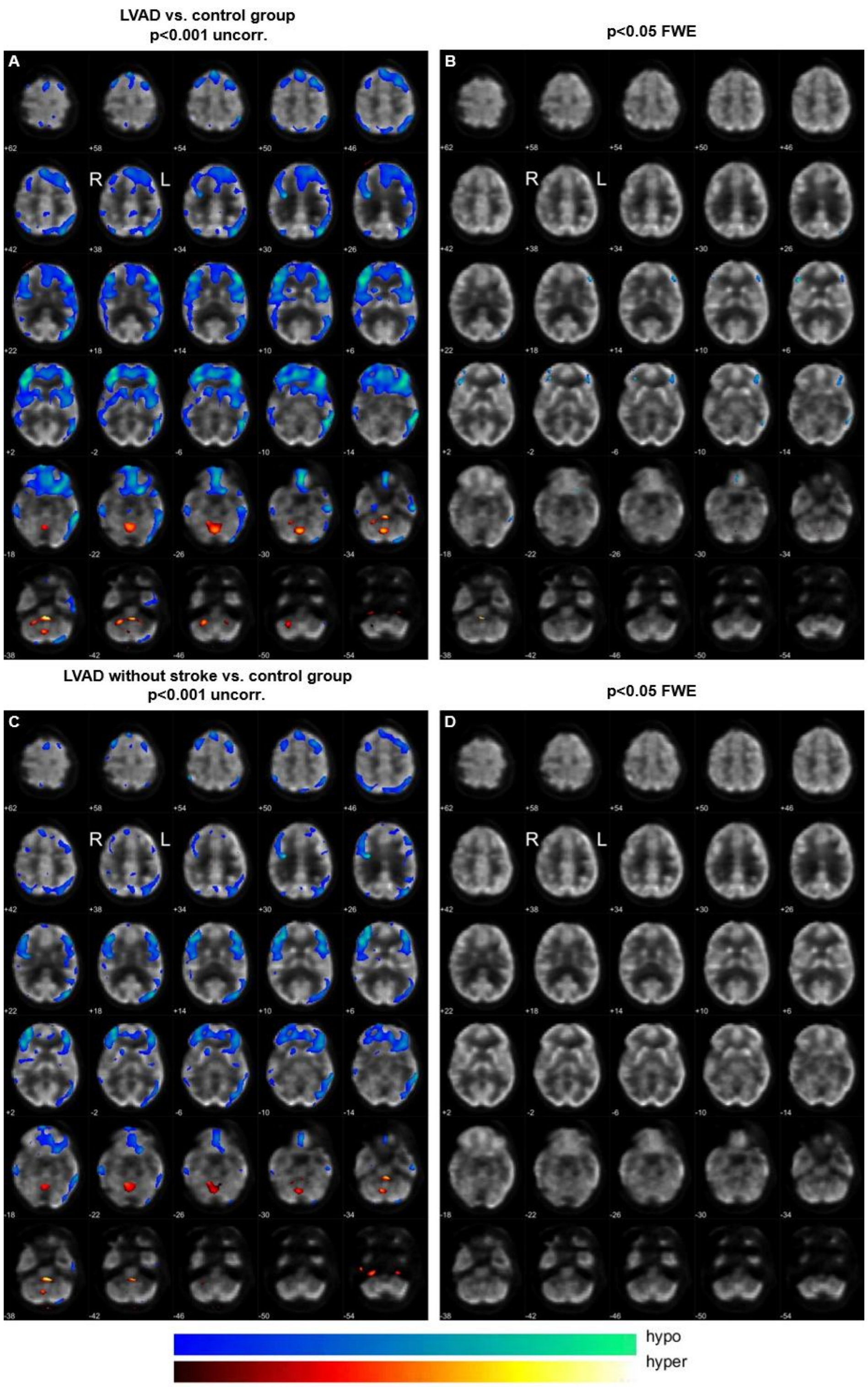

(A) A p<0.001 uncorrected for multiple testing was applied to the analysis showing a widespread, frontotemporally accentuated hypometabolism. (C) Analyzing all LVAD patients

without a history of stroke ($n = 10$) reduced the extent of hypometabolism but proved the frontal focus. (B+D) Application of $p<0.05$ with FWE correction resulted in extensive reduction of significant hypometabolism in the conducted group comparisons. Abbreviations: LVAD Left ventricular assist device, FWE family-wise error, SPM single-subject statistical parametric mapping, ^{18}F-FDG-PET/CT 18F-fluordeoxyglucose positron emission tomography/computed tomography

Figure S2: Combined SPM results for hypo- and hypermetabolic contrast of SSVCG analyses in ^{18}F-FDG-PET/CT as well as MRI FLAIR series exemplarily from one HM3 patient.

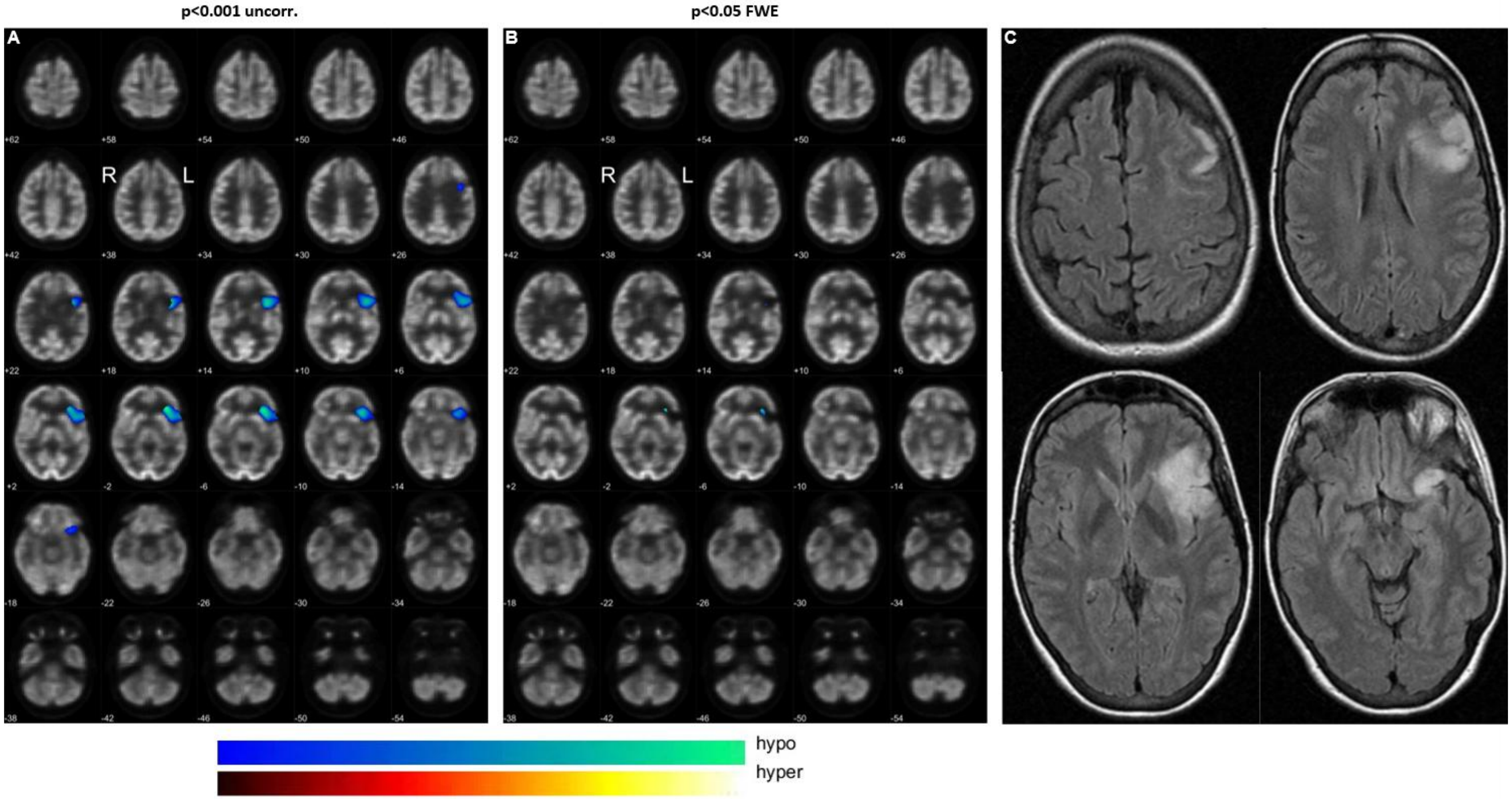

(A) The SSVCG analysis was performed using a P-value < 0.001 uncorrected for multiple testing showing primarily a hypometabolic cluster in the left frontal lobe consistent with the location of a suffered stroke. (B) SSVCG analysis using a P-value <0.05 FWE corrected showed extensive reduction in significant hypometabolism compared to p <0.001 uncorr.. Most of the infarct core (i.e. the black wedge in the left frontal lobe in the PET images) is missing significant hypometabolism (C) MRI FLAIR images showing an infarct core in the left frontal and temporal lobe. Abbreviations: SPM single-subject statistical parametric mapping, SSVCG single-subject vs. control group, ^{18}F-FDG-PET/CT ^{18}F-fluorodeoxyglucose (FDG)-positron emission tomography/computed tomography, MRI magnetic resonance imaging, FLAIR fluid-attenuated inversion recovery, HM3 Heart Mate 3, FWE family-wise error
